# To Eat and to Be Eaten: Mutual Metabolic Adaptations of Immune Cells and Intracellular Bacterial Pathogens upon Infection

**DOI:** 10.3389/fcimb.2017.00316

**Published:** 2017-07-13

**Authors:** Wolfgang Eisenreich, Thomas Rudel, Jürgen Heesemann, Werner Goebel

**Affiliations:** ^1^Department of Chemistry, Chair of Biochemistry, Technische Universität München Garching, Germany; ^2^Department of Microbiology, Biocenter, University of Würzburg Würzburg, Germany; ^3^Max von Pettenkofer-Institute, Chair of Medical Microbiology and Hospital Epidemiology, Ludwig Maximilian University of Munich München, Germany

**Keywords:** pathometabolism, immunometabolism, innate immune system, metabolic adaptation, intracellular pathogens

## Abstract

Intracellular bacterial pathogens (IBPs) invade and replicate in different cell types including immune cells, in particular of the innate immune system (IIS) during infection in the acute phase. However, immune cells primarily function as essential players in the highly effective and integrated host defense systems comprising the IIS and the adaptive immune system (AIS), which cooperatively protect the host against invading microbes including IBPs. As countermeasures, the bacterial pathogens (and in particular the IBPs) have developed strategies to evade or reprogram the IIS at various steps. The intracellular replication capacity and the anti-immune defense responses of the IBP's as well as the specific antimicrobial responses of the immune cells of the innate and the AIS depend on specific metabolic programs of the IBPs and their host cells. The metabolic programs of the immune cells supporting or counteracting replication of the IBPs appear to be mutually exclusive. Indeed, recent studies show that upon interaction of naïve, metabolically quiescent immune cells with IBPs, different metabolic activation processes occur which may result in the provision of a survival and replication niche for the pathogen or its eradication. It is therefore likely that within a possible host cell population subsets exist that are metabolically programmed for pro- or anti-microbial conditions. These metabolic programs may be triggered by the interactions between different bacterial agonistic components and host cell receptors. In this review, we summarize the current status in the field and discuss metabolic adaptation processes within immune cells of the IIS and the IBPs that support or restrict the intracellular replication of the pathogens.

## Introduction

Metabolism governs virtually all dynamic processes of living cells. A functioning metabolism requires sufficient supply of basic nutrients, including carbon-, nitrogen-, and sulfur-sources, for (a) the generation of energy in form of ATP, (b) the production of low-molecular and high-molecular cellular building blocks, and (c) the maintenance of an intracellular redox status.

For achieving these three goals, prokaryotic microorganisms have evolved many different autotrophic and heterotrophic metabolic strategies including assimilation of carbon dioxide, specific transport systems and catabolic pathways for many different carbon sources, a large number of anabolic pathways, various aerobic and anaerobic ATP-generating reactions and the adaptation to a wide range of temperature, pH and pressure.

Mammalian cells, on the other hand, carry out a rather common type of heterotrophic metabolism which supports cell growth and proliferation through catabolism of a limited number of carbon sources, mainly glucose, fatty acids and amino acids (especially glutamine), thereby gaining (a) energy (ATP) preferentially by oxidative phosphorylation (OXPHOS, for a list of all abbreviations, see above and the Supplementary Table) via aerobic respiration and/or by substrate phosphorylation mainly via glycolysis and (b) intermediates for a limited number of anabolic processes (Ghesquiere et al., 2014). This metabolism functions optimally at mesophilic temperature, close to neutral pH and (micro)aerophilic conditions and is controlled (similar to prokaryotic cells) by a complex network of signaling pathways, transcription factors, microRNAs and post-transcriptional mechanisms (Storey and Storey, 2004; Storey, 2015) (for more details, see below). Most differentiated mammalian cells can reversibly switch from a quiescent metabolic state characterized by low catabolic and anabolic activity and OXPHOS (Palsson-Mcdermott and O'Neill, 2013) to a proliferating state with high catabolic and (often) high anabolic activities. This activated metabolism requires fast energy (mainly ATP) generation often driven by “aerobic glycolysis” (also known as “Warburg effect”).

Most bacterial pathogens that are able to colonize and grow in mammalian niches are optimally adapted to a mesophilic and heterotrophic metabolic life style. This is especially true for all human intracellular bacterial pathogens (IBPs) which are characterized by the ability to invade and proliferate in different mammalian cell types (Fuchs et al., 2012), including professional phagocytes of the innate immune system (IIS), in particular macrophages (MPs), dendritic cells (DCs) and (less frequently) neutrophilic polymorphonuclear leukocytes (PMNs), or neutrophils (NPs). Uptake of the IBPs by these cells occurs by receptor-triggered phagocytosis involving e.g., Fc-γ receptors, scavenger receptors, integrin receptors or C-type lectin receptors (Drummond and Brown, 2013; Freeman and Grinstein, 2014). Internalization of IBPs by non-professional phagocytes, e.g., epithelial and endothelial cells, requires the interaction between specific bacterial invasion factors and appropriate host cell receptors which triggers internalization (Pizarro-Cerdá and Cossart, 2006).

Once internalized, the IBPs replicate within the host cells in specialized vacuoles, in the cytosol or in both compartments (Knodler, 2015). Successful replication of the IBPs in these compartments requires adaptation of the bacteria to the metabolism of host cells which in turn may be reprogrammed by the infection with IBPs. Since the metabolic potentials of the IBPs significantly differ, the metabolic adaptation of IBPs to their host cells must require different, yet still poorly understood strategies on part of the IBPs and the host cells (Eisenreich et al., 2010; Fuchs et al., 2012; Abu Kwaik and Bumann, 2015).

For the protection against microbial pathogens, the mammalian hosts have evolved a highly efficient integrated immune system, consisting of the IIS and the adaptive immune system (AIS) which cooperatively interact. The professional phagocytes as well as the mucosal epithelial cells and the skin keratinocytes belonging to the IIS, and also the primed and activated lymphocytes of the AIS possess potent antimicrobial capacities against invading pathogens, including IBPs.

On the other hand, most of the immune cells, especially those of the IIS, may also serve as proficient host cells for IBPs. Compared to the hospitality of IIS immune cells, the capacity of the immune cells of the AIS, i.e., T and B lymphocytes, to function as host cells for IBPs appears to be limited (Menon et al., 2003; Geddes et al., 2007; Krocova et al., 2008; Konradt et al., 2011; Goenka et al., 2012; Nothelfer et al., 2014).

It is an apparent paradox that the same cell types of the IIS may represent convenient replication niches for the IBPs but also the most important tools for the destruction of IBPs. The immune (defense) responses triggered by IBPs have been extensively studied while the metabolic aspect has been rather neglected (Price and Vance, 2014; O'Neill and Pearce, 2016). In this review, we will therefore mainly focus on this latter aspect with emphasis on IBP-infected immune cells of the IIS.

There is growing evidence that both, the efficient expression of the antimicrobial activities of the immune cells and the successful replication of IBPs within these cells require specific metabolic programs in both interacting partners for which the terms “immunometabolism” (Rathmell, 2012; Pollizzi and Powell, 2014) and “pathometabolism” (Eisenreich et al., 2015), respectively, have been coined. These two metabolic entities seem to mutually influence each other when the immune cells meet IBPs.

In the first part of this review, we provide some relevant background information on the metabolism and the regulatory networks of IBPs and of mammalian host cells in general, however with a focus on the immune cells of the IIS. In the second part, we discuss the present, but still rather limited knowledge concerning the metabolic adaptation processes which occur in both partners during the interaction of IBPs with these immune cells.

## Metabolic characteristics of intracellular bacterial pathogens and their mammalian host cells

### Intracellular bacterial pathogens (IBPs)

**Figure 1:**
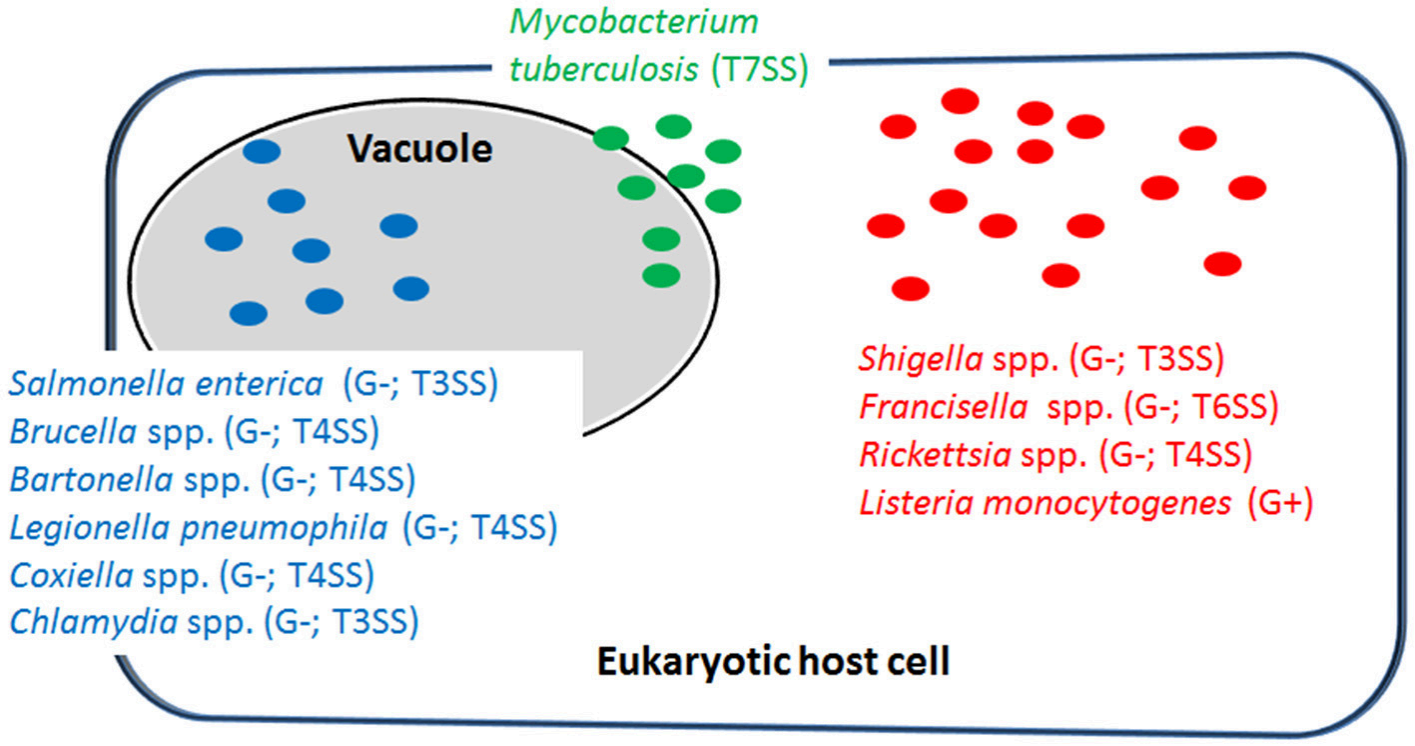
Representative examples of intracellular bacterial pathogens (IBPs; mentioned in this review) replicating in specialized vacuoles (in blue) or in the host cell's cytosol (in red). Some IBPs (e.g., *M. tuberculosis*) are able to replicate in both cellular compartments (in green). G−, Gram-negative; G+, Gram-positive; T3SS, type 3-secretion system; T4SS, type 4-secretion system; T6SS, type 6-secretion system; T7SS, type 7-secretion system.

Intracellular bacterial pathogens (IBPs) represent a group of disease-causing bacteria that are able to invade a variety of differentiated mammalian cells of various differentiation states, including immune cells (in particular those of the IIS), and to survive and proliferate in specialized membrane-surrounded vacuoles or in the cytosol (Figure 1) for extended periods of time before leaving the host cells by cell disruption or cell-to-cell spread (Cossart and Sansonetti, 2004; Kumar and Valdivia, 2009; Ray et al., 2009; Fredlund and Enninga, 2014). Most of the IBPs are facultative intracellular, i.e., they are able to replicate within host cells, but also extracellularly in tissues or mucosal excretions, in natural environments and in more or less complex cell-free (“axenic”) media (Omsland et al., 2009). The efficient replication of a minority of IBPs (so-called “obligate IBPs”) appears to be strictly dependent on host cells (Wood et al., 2014).

Most IBPs have a typical Gram-negative cell envelope structure and inject (often many) effector proteins by specific protein secretion/injection systems (mainly type 3, and 6 secretion systems, T3SS, T4SS or T6SS; see Figure 1) into the host cells cytosol (Costa et al., 2015). Most of these effectors, whose functions are currently intensively investigated, are apparently involved in the uptake and/or the survival/proliferation processes of these IBPs by interfering mainly with different signaling pathways of host cells affecting cytoskeletal dynamics, survival, innate immune responses and possibly also metabolism of the contacted host cells (Alto and Orth, 2012; Eisenreich et al., 2013). The best-studied representative among the less frequently occurring Gram-positive IBPs is *Listeria monocytogenes* that replicates in the cytosol of infected host cells. The pathogenic *Mycobacterium* species have a unique cell envelope different from the typical cell envelopes of Gram-positive and Gram-negative bacteria. These pathogens possess type 7 protein secretion systems (ESX/T7SS) that secrete proteins some of which are clearly involved in pathogenicity and host cell interaction (Simeone et al., 2009; Houben et al., 2014).

Numerous virulence factors essential for invasion, intracellular survival and proliferation of the IBPs have been characterized (Cossart and Sansonetti, 2004). Although *L. monocytogenes* as Gram-positive pathogen lacks a protein injection apparatus, it produces a variety of secreted and cell-bound internalins that interact as ligands with different host cell receptors thereby performing in part similar trigger functions as the Gram-negative effector proteins (Bierne et al., 2007; Mcgann et al., 2007).

**Figure 2:**
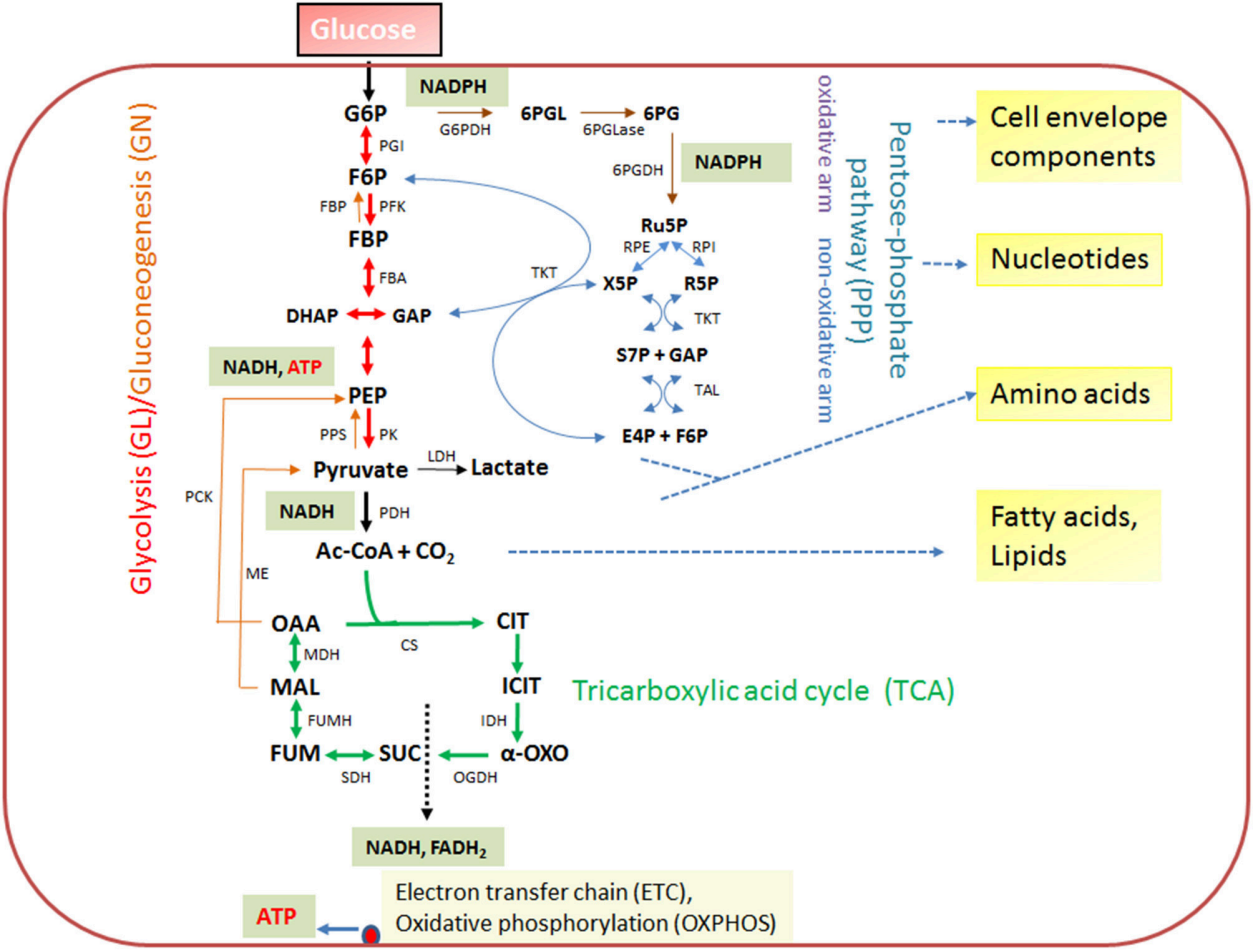
Schematic presentation of the basic catabolic and anabolic processes of a typical heterotrophic prokaryotic cell: glycolytic pathway (GL), pentose-phosphate pathway (PPP) with its oxidative and non-oxidative arms, and tricarboxylic acid cycle (TCA). The specific reactions essential for gluconeogenesis (GN) (in addition to the reversible GL reactions) are catalyzed by PEP carboxykinase (PCK), PEP synthetase (PPS) and fructose-1,6-diphosphatase (FBPase). The anaplerotic reactions are not shown with the exception of the reaction leading from malate to pyruvate, catalyzed by the malic enzyme (ME) which may play an important role the intracellular metabolism of IBPs (see Figure 3). The ATP production by oxidative phosphorylation (OXPHOS) via the electron transfer chain (ETC) is also indicated. Metabolites of GL: G6P, glucose-6P; F6P, fructose-6P; FBP, fructose-1,6 diphosphate; DHAP, dihydroxyacetone phosphate; GAP, glyceraldehyde-3P; PEP, phosphoenolpyruvate. Metabolites of the PPP: 6PGL, 6-phosphogluconolactone; 6PG, 6-phosphogluconate; Ru5P, ribulose-5P; X5P, xylulose-5P; R5P, ribose-5P; S7P, sedoheptulose-7P; E4P, erythrose-4P. Metabolites of the tricarboxylic acid cycle (TCA): OAA, oxaloacetate; CIT, citrate; ICIT, isocitrate; α-OXO, alpha-oxoglutarate; SUC, succinate, FUM, fumarate, MAL, malate. Enzymes of the GL: PGI, phosphoglucoisomerase; PFK, phosphofructokinase; FBA, fructobisphosphate aldolase; PK, pyruvatekinase. PDH, pyuvate dehydrogenase. LDH, lactate dehydrogenase. Enzymes of the PPP: G6PDH, glucose-6P dehydrogenase; 6PGLase, 6-phosphogluconolactonase; 6PGDH, 6-phosphogluconate dehydrogenase; RPE, Ru5P-epimerase; RPI, Ru5P-isomerase; TKT, transketolase; TAL, transaldolase. Enzymes of the TCA cycle: CS, citrate synthase; IDH, isocitrate dehydogenase; OGDH, oxoglutarate dehydrogenase; SDH, succinate dehydrogenase; FUMH, fumarate hydratase; MDH, malate dehydrogenase.

**Figure 3:**
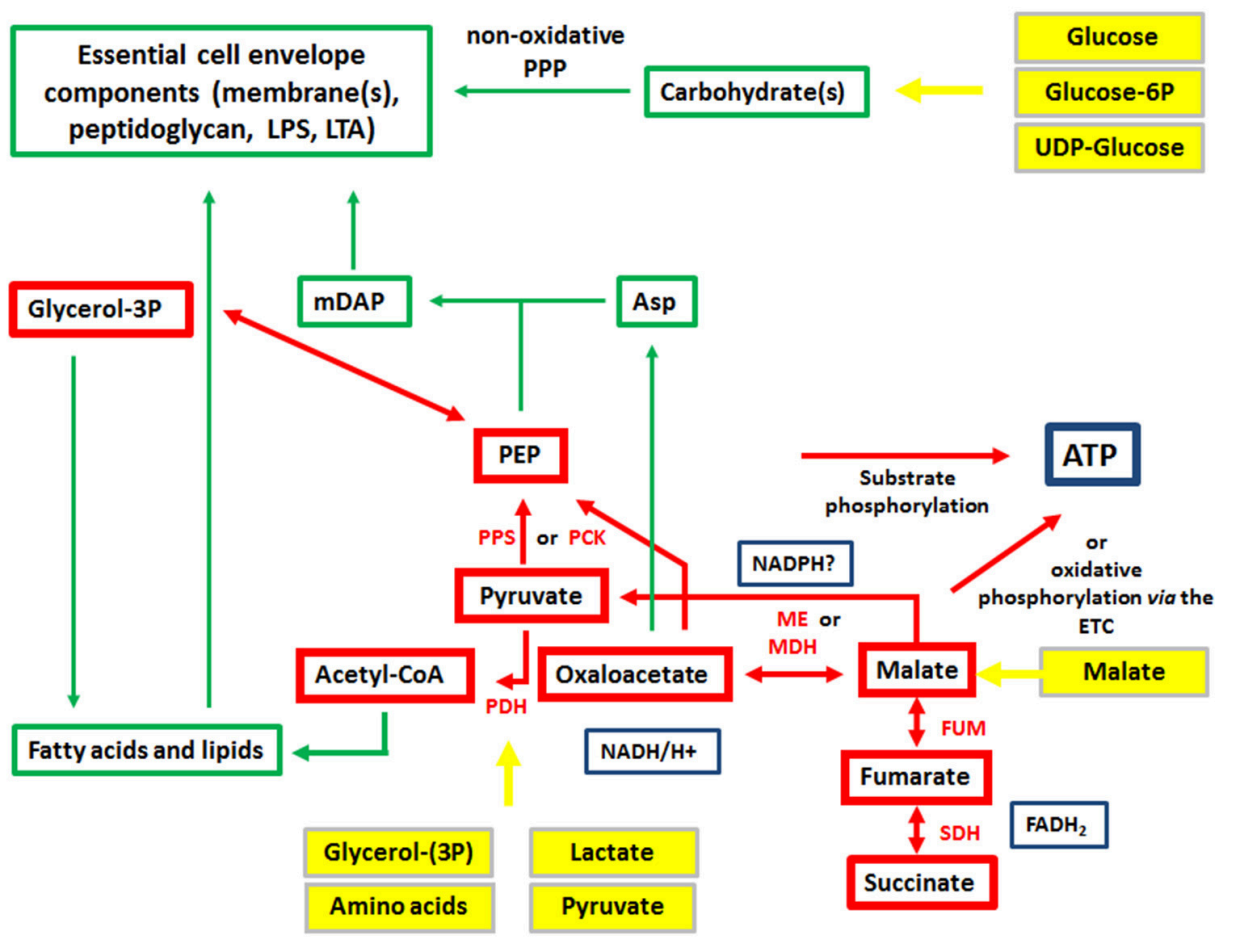
Metabolism of IBPs: metabolites and metabolic reactions common to all IBPs. Red boxes and arrows show the common catabolic products and reactions, respectively and green boxes and arrows the common anabolic products and reactions. Produced co-factors are in blue-framed boxes. Products in yellow boxes and arrows represent nutrients that can be delivered by the host cell.

The expression of the major virulence genes of IBPs is often controlled by master transcription regulators, with links to the metabolism of the IBP and even of the host cell (Stoll et al., 2008; Poncet et al., 2009; De Las Heras et al., 2011; Gillmaier et al., 2012; Reniere et al., 2015). Not surprisingly, all IBPs are heterotrophic, aerobic or facultative anaerobic bacteria, able to survive and replicate under the normoxic and hypoxic conditions which they may encounter during their infection cycles. The metabolic potentials of most IBPs are reduced compared to the metabolic capacity of typical heterotrophic generalists (e.g., *E. coli;* see Figure 2). The extent of the metabolic reduction differs, however, strongly among these IBPs in view of the catabolic as well as the anabolic capabilities (Eisenreich et al., 2010; Fuchs et al., 2012), suggesting that the dependency on nutrient supply to be delivered by the host cell and the adaptation of the bacterial metabolism to that of the host cell must be specific for each IBP. Interestingly, there are, however some metabolic functions that are maintained by all IBPs and hence might be indispensable for intracellular bacterial survival (Figure 3).

The metabolic burden put on the host cell by these IBP infections has a strong impact on the host cells metabolism, causing activation of specific signaling pathways which may result in increased catabolic, energy and anabolic metabolism but also in induction of apoptosis/pyroptosis and autophagy (Eisenreich et al., 2013). These metabolic changes may in turn influence the metabolism of the intracellular bacteria. Thus, a complex network of metabolic interactions between the IBPs and host cells exists which determines the fate of IBPs within host cells, especially immune cells of the IIS (Eisenreich et al., 2015).

### The mammalian host cells

**Figure 4:**
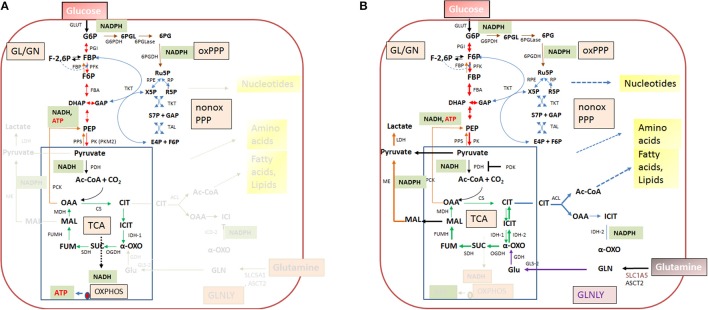
Quiescent **(A)** and activated **(B)** metabolism in differentiated mammalian cells. In differentiated mammalian cells, including mucosal epithelial cells (MEC) and non-activated immune cells, the metabolism is characterized by low catabolic and anabolic activities. Low carbon fluxes fed by glucose and/or fatty acids run through glycolysis, PPP, and the TCA cycle; oxidative phosphorylation (OXPHOS) is the predominant source of ATP. The metabolism of metabolically activated cells (B) is characterized by enhanced glucose uptake, highly activated (“aerobic”) glycolysis, reduced entrance of pyruvate into the TCA cycle and reduced OXPHOS. ATP is mainly produced by substrate phosphorylation in the glycolytic pathway. In addition these cells show often increased glutaminolysis which replenishes the TCA cycle, leading to citrate (eventually via reverse IDH) which is transported into the cytosol where it is converted by the ATP-dependent citrate lyase (ACL) into oxaloacetate and acetyl-CoA, two metabolites serving for biosynthesis of fatty acids/lipids and amino acids. ß-oxidation is inhibited. Metabolites of GL: G6P, glucose-6P; F6P, fructose-6P; FBP, fructose-1,6 biphosphate; DHAP, dihydroxyacetonephosphate; GAP, glyceraldehyde-3P; PEP, phosphoenolpyruvate. Metabolites of the PPP: 6PGL, 6-phosphogluconolactone; 6PG, 6-phosphogluconate; Ru5P, ribulose-5P; X5P, xylulose-5P; R5P, ribose-5P; S7P, sedoheptulose-7P; E4P, erythrose-4P. Metabolites of the tricarboxylic acid (TCA) cycle: OAA, oxaloacetate; CIT, citrate; ICIT, Isocitrate; α-OXO, alpha-oxoglutarate; SUC, succinate, FUM, fumarate, MAL, malate. Enzymes of the GL: GLUT, glucose transporter; PGI, phosphoglucoisomerase; PFK, phosphofructokinase; FBA, fructobisphosphate aldolase; PK, pyruvatekinase. PDH, pyuvate dehydrogenase. LDH, lactate dehydrogenase. Enzymes of the PPP: G6PDH, glucose-6P dehydrogenase; 6PGLase, 6-phosphogluconolactonase; 6PGDH, 6-phosphogluconate dehydrogenase; RPE, Ru5P-epimerase; RPI, Ru5P-isomerase; TKT, transketolase; TAL, transaldolase. Enzymes of the TCA cycle: CS, citrate synthase; ICD, isocitratedehydogenase; OGDH, oxoglutarate dehydrogenase; SDH, succinate dehydrogenase; FUMH, fumarate hydratase; MDH, malate dehydrogenase. Glutaminolysis (GLNLY): SLC1A5, ASCT2, glutamine transporters, GLS, glutaminase; GDH, glutamate dehydrogenase.

In contrast to the highly diverse metabolic potentials of the IBPs, the metabolic equipment of mammalian cells is quite similar (Figure 4). But in spite of this, the metabolic activities of host cells can change dramatically depending on the differentiation state, external and internal signals and (in case of cancer cells) genetic alterations.

Without external stimuli (e.g., growth factors) most terminally differentiated mammalian cells, including the immune cells of the IIS are non-proliferating or only slowly proliferating, i.e., these cells are in a (more or less) metabolically quiescent state (Figure 4A). In this state they take up, at a low rate, glucose—dependent on the cell type—by one of several glucose transporters (GLUTs) (Chen et al., 2015). Glucose is then phosphorylated by hexokinase (HK) to glucose-6-phosphate (G6P) and further metabolized in the cytosol through the glycolytic pathway (GL) and/or the pentose phosphate pathway (PPP). G6P is oxidized in the GL to pyruvate which is shuttled into mitochondria where it is further oxidized to carbon dioxide by pyruvate dehydrogenase (PDH) and the tricarboxylic acid cycle (TCA), generating NADH/H^+^, FADH_2_, GTP, and intermediates, essential for anabolic pathways (especially fatty acids/lipids, amino acids). G6P can be also oxidized by G6P dehydrogenase (G6PDH) and 6-phosphogluconate dehydrogenase (6PGDH) to ribulose-5P (Ru5P) in the oxidative branch of the PPP. Both reactions generate NADPH, an important co-factor for reductive biosynthetic pathways (e.g., fatty acids/lipids, deoxyribonucleotides) and scavenging of reactive oxygen species (ROS). In the non-oxidative branch of the PPP, Ru5P is reversibly converted by ribose-5-phosphate isomerase (RPI) to ribose-5P (R5P) which is an essential precursor to many biomolecules, especially nucleotides, and to xylulose-5P (X5P) by ribulose-5-phosphate 3-epimerase (RPE). This non-oxidative PPP branch consists of additional reversible reactions catalyzed by transketolase (TKT) and transaldolase (TAL) and recruits the glycolytic intermediates fructose-6P (F6P) and glyceraldehyde-3P (G3P), which can thereby also be converted into pentose phosphates (and *vice versa*) thus linking GL and PPP (see Figure 4). Indeed, GL and PPP are coordinately regulated to support cell growth and survival as discussed below in more detail.

Under non-activated cell conditions, ATP production occurs mainly by OXPHOS in the electron transport chain (ETC) whereby the electrons of NADH/H+ (generated in GL and TCA cycle) are transferred to oxygen as final acceptor. Under these normal physiological conditions growth factors are available only in limited and strictly controlled amounts (Rathmell et al., 2000). Hence, most terminally differentiated cells perform a catabolic metabolism which allows optimal efficiency of ATP production from limited nutrient supply (DeBerardinis et al., 2006; Vander Heiden et al., 2009; Ward and Thompson, 2012).

When growth factor concentration is increased, nutrient uptake (most notably by glucose and glutamine transporters) is induced and the intracellular nutrient pools rise. These cells now adopt a more anabolic metabolism (i.e., enhanced biosynthesis of nucleotides, “non-essential” amino acids, fatty acids/lipids and the macromolecules), double the cellular biomass and initiate cell division (DeBerardinis et al., 2008; Vander Heiden et al., 2009). Compared to the non-activated differentiated cells, the growth factor-stimulated, proliferating cells do not optimize efficiency of ATP production but rather the production of larger amounts of intermediary metabolites and NADPH for glutathione production (essential for antioxidant defense) and other biosynthetic reactions (Figure 4B). ATP is now mainly produced through glycolysis by substrate phosphorylation. Excess pyruvate generated as final product in the GL is reduced to lactate which is secreted. The reaction also restores NAD necessary for the maintenance of the glycolytic flux. This kind of metabolism (“aerobic glycolysis”), which is energetically inefficient but leads to fast ATP production and favors anabolic processes, is characteristic for many fast proliferating cancer cells (DeBerardinis, 2008; Ngo et al., 2015). Although the cells of the mononuclear phagocyte system (MPS), comprising monocytes (MOs), MPs and DCs, do not proliferate in tissue after extravasation, they can switch their metabolism upon transition from the quiescent to the activated state. The metabolism in the activated state of MP cells shows similarities to that of cancer cells (O'Neill and Pearce, 2016).

### Oncogenes and tumor suppressors as important regulators of metabolic pathways

**Figure 5:**
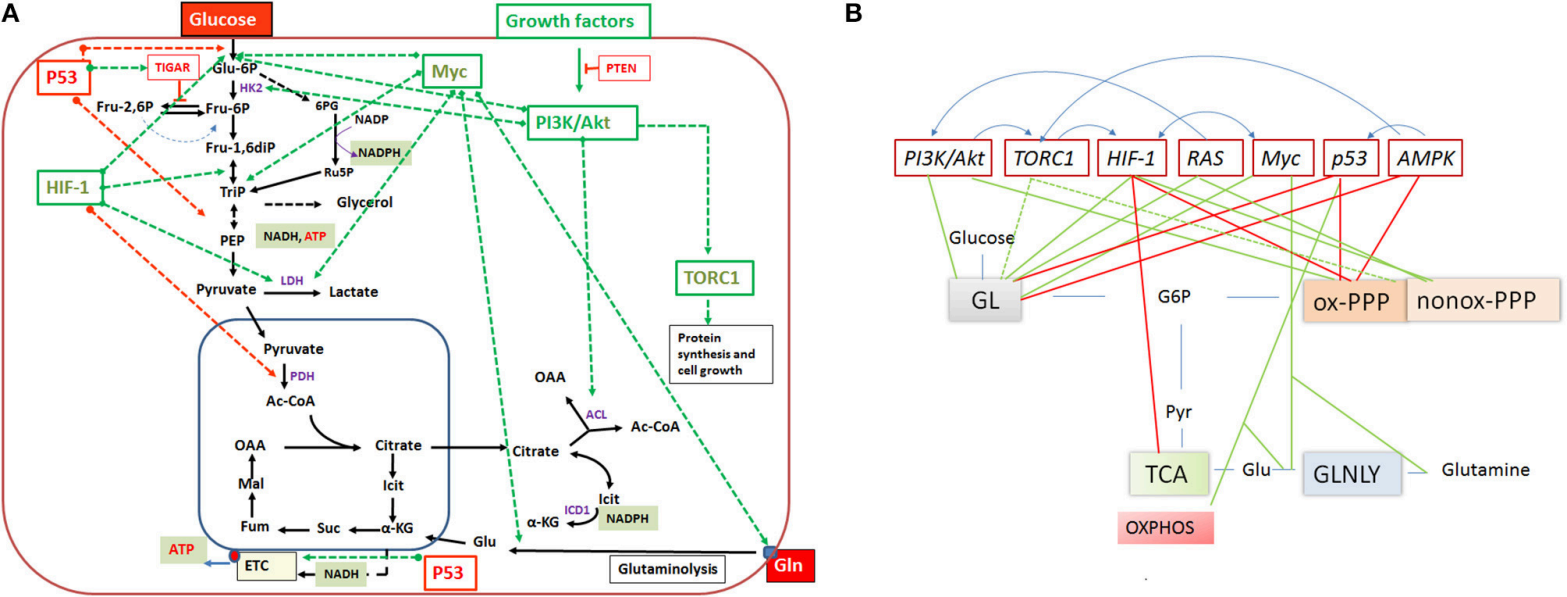
**(A)** Important regulators of the mammalian cell metabolism are (proto)-oncogenes and tumor suppressors and interacting regulatory factors (e.g., PI3K/AKT, RAS, Myc, TORC1, HIF-1 and P53, PTEN, TIGAR, respectively) that control key reactions of the central catabolic pathways (including glutaminolysis) either positively (green dashed arrows) or negatively (red dashed arrows). **(B)** A schematic overview on the catabolic pathways including glycolysis (GL), pentose phosphate pathway (PPP), tricarboxylic acid (TCA) cycle and glutaminolysis (GLNLY) controlled by these key regulators. Green lines indicate upregulation of catabolic pathways and red lines downregulation of the pathways. Anabolic pathways (i.e., those leading to synthesis of nucleotides, amino acids, glutathione, lipids) which depend on intermediates of the catabolic pathways are (indirectly) also influenced by these regulators. The blue arrows above the regulators indicate interactions between them. See text for further details.

Expression of the pathways involved in carbon and energy metabolism of mammalian cells is controlled by a complex net of nutrient sensors, growth hormone receptors, several downstream signaling pathways, canonical transcription factors including NF-κB, AP-1, NFAT, ChREBP, PPARα, SREBP as well as non-coding RNAs (Eberle et al., 2004; Postic et al., 2007; Fritz and Fajas, 2010; Cairns et al., 2011; Thompson, 2011; Harris et al., 2012; Iizuka et al., 2013; Ganeshan and Chawla, 2014; Jiang et al., 2014; Pollizzi and Powell, 2014; Yu et al., 2015). In the last decade, studies in particular on the dysregulated metabolism of cancer cells also highlighted the significance of (proto)oncogenes and tumor suppressors as key regulators of cellular metabolism. Most important players are here PI3K/AKT1, mTORC1, AMPK, RAS, HIF-1, cMYC, and p53 (Figure 5). Whereas, the impact of these factors on the regulation of growth cycle, differentiation, proliferation, immune responses, survival, and apoptosis of mammalian cells were already extensively investigated in the past, their involvement in the regulation of central metabolic pathways was recognized only recently. For recent reviews, see (Yin et al., 2012; Iurlaro et al., 2014). In the following, we focus mainly on the impact of the latter factors for the regulation of the central carbon metabolism, since they may be primary targets for the interaction with specific effector proteins of IBPs resulting in the reprogramming of the host cell metabolism during infection (Table 1).

**Table 1:** Interactions of IBP effectors with their host cell targets.

**IBP**	**Bacterial effector**	**Host cell target**	**References**
*Salmonella typhimurium*	AvrA	p53	Wu et al., 2010
*Salmonella typhimurium*	SopB	Akt	Cooper et al., 2011; Roppenser et al., 2013
*Salmonella typhimurium*	Unknown	AMPK	Arsenault et al., 2013
*Salmonella enterica*	Salmochelin	HIF-1α	Hartmann et al., 2008
*Shigella flexneri*	VirA	Calpain/p53	Bergounioux et al., 2012
*Shigella flexneri*	IpgD	PI3K/Akt	Pendaries et al., 2006; Ramel et al., 2009
*Shigella flexneri*	OspF	MAPK, JNK	Reiterer et al., 2011
*Chlamydia trachomatis*	Unknown	p53	Siegl et al., 2014
*Chlamydia trachomatis*	Unknown	EphA2/PI3K/Akt	Subbarayal et al., 2015
*Chlamydia pneumoniae*	CPAF	HIF-1α	Rupp et al., 2007
*Brucella melitensis*	Unknown	p53	Liu et al., 2012
*Legionella pneumophila*	Unknown	PI3K/Akt	Tachado et al., 2008
*Legionella pneumophila*	Unknown	AMPK	Francione et al., 2009
*Listeria monocytogenes*	Unknown	PI3K/Akt	Tachibana et al., 2015
*Francisella tularensis*	Unknown	mTOR	Edwards et al., 2013
*Mycobacterium tuberculosis*	Unknown	PTEN	Huang et al., 2012
*Mycobacterium tuberculosis*	Mtb RNA-TLR3	PI3K/Akt	Bai et al., 2014
*Mycobacterium tuberculosis*	ESAT-6	PI3K/MAPK	A et al., 2012
*Mycobacterium tuberculosis*	Unknown	p53	Galietti et al., 2001

#### PI3K/Akt

The major signaling pathway downstream of growth factor receptors activating glucose flux is the phosphoinositide-3-kinase/Akt (PI3K/Akt) pathway (Hemmings and Restuccia, 2012). This pathway is also one of the most commonly altered signaling pathways in human cancer cells. PI3K generates phosphatidylinositol-3,4,5-trisphosphate (PIP3) from phosphatidylinositol-4,5-bisphosphate (PIP2) and PIP3 recruits Akt to the cell membrane permitting its activation by upstream kinases (e.g., 3-phosphoinositide dependent protein kinase-1 (PDPK1). The activated Akt then stimulates expression of several proteins (Figure 5) involved in glucose uptake and glycolysis (DeBerardinis et al., 2008), including the glucose transporter 1 (GLUT1) (through enhanced translation and translocation to the cell surface), hexokinase 2 (HK2), and phosphofructokinase 1 (PFK1). In addition to its regulatory function in glycolysis, the PI3K-Akt pathway may regulate G6PDH and thus the oxidative arm of PPP (Wagle et al., 1998). Furthermore, Akt activates ATP-dependent citrate lyase (ACL). This cytosolic enzyme cleaves citrate (exported from the mitochondria into the cytosol) into oxaloacetate and acetyl-CoA, thereby providing essential precursors for aspartate and fatty acid/lipid biosynthesis, respectively. Finally, it is noteworthy that chemokine-triggered migration of invasive cells involves the PI3K/Akt pathway.

#### mTORC1

The target of rapamycin (mTOR) complex 1 (mTORC1) is an important regulator of metabolism downstream of PI3K-Akt and is often constitutively activated during tumorigenesis (Guertin and Sabatini, 2007). Phosphorylated Akt activates mTORC1 by direct phosphorylation and by inactivation of the tuberous sclerosis protein 2 (TSC2), an inhibitor of mTORC1. A genomic approach (Duvel et al., 2010) suggests that mTORC1 induces transcription of several genes involved in glycolysis and both branches of PPP. The molecular nature of the activation of these genes by mTORC1 remains, however, elusive. The activity of this complex is also modulated by sensing essential amino acids (especially leucine) and hence represents a central regulator of amino acid metabolism. In response to these signals, mTORC1 positively regulates protein biosynthesis by activation of S6 kinase (S6K) which enhances expression of ribosomal proteins and by inhibiting the initiation factor 4E-binding protein (4E-BP). The mTOR complex 2 (mTORC2) lies upstream of Akt, is positively regulated by PI3K signaling and is also involved in the activation of mTORC1 (Liu et al., 2013).

#### Ras

The Ras proteins (RAS including K-Ras, H-Ras, and N-Ras) are low molecular weight G proteins whose activity is regulated by binding of guanine nucleotides. In the activated GTP-bound state, RAS modulates a number of signaling pathways. The two best-studied downstream pathways that are induced by activated RAS are the mitogen-activated protein kinases (MAPK) and the PI3K/Akt pathways (Gysin et al., 2011). Oncogenic RAS mutations, leading to loss of GTPase activity, tend to lock RAS in the active (GTP-bound) state which results in constitutive RAS signaling (Pratilas and Solit, 2010). As shown by Telang and colleagues (Telang et al., 2007), activated H-Ras increases in immortalized human bronchial epithelial cells, the glycolytic flux to lactate, TCA cycle activity and oxygen consumption. The same group showed that activation of H-Ras enhances cytochrome C oxidase (COX) activity and mitochondrial respiration in part *via* up-regulation of the COX Vb subunit (Telang et al., 2012; Chesney and Telang, 2013). Based on these data, it was postulated that H-Ras activation may cause simultaneous stimulation of COX activity and uncoupling of ATP synthase from the proton gradient in order to facilitate the continued oxidation of NADH to NAD necessary for glycolytic flux and TCA cycling.

In pancreatic tumors, oncogenic K-Ras caused stimulation of glucose uptake and channeling of glycolysis intermediates into the non-oxidative arm of the PPP, thereby promoting ribose-5P biogenesis and decoupling it from NADPH production (Ying et al., 2012). The oxidative branch of PPP and the TCA cycle were unaffected by the activated K-Ras. The authors further showed that the MAPK pathway and Myc-directed transcriptional control but not the PI3K/Akt pathway played the crucial role for the K-Ras-mediated metabolic reprogramming in these tumors.

#### HIF-1

The hypoxia-inducible transcription factor 1 (HIF-1), a heterodimer consisting of the HIF-1α and HIF-1β subunits (Iyer et al., 1998), plays also a central role in the regulation of cellular glucose catabolism. Whereas, HIF-1β is stable and constitutively expressed, expression of HIF-1α is induced and its active form is stabilized under low cellular oxygen (hypoxic) conditions. The stability of HIF-1α is regulated by prolyl hydroxylase (PHD)-mediated hydroxylation of proline residues of the HIF-1α subunit. This reaction targets HIF-1α for its recognition by the ubiquitin E3 ligase and its subsequent proteasomal degradation. Oxygen is required for the PHD activity. However, at low oxygen and in the presence of elevated levels of ROS or the two TCA intermediates, succinate and fumarate, PHD activity and hence HIF-1α degradation are inhibited. But even under normoxic conditions, HIF-1 can be positively regulated (on the transcriptional and the translational level) downstream of growth factor receptors by the PI3K/Akt/mTOR pathway (Dery et al., 2005; Lisy and Peet, 2008; Kuschel et al., 2012; Burroughs et al., 2013).

HIF-1 enhances transcription of genes encoding GLUTs and most enzymes of the glycolytic pathway. HIF-1 activation may also lead to a reduced flux through the oxidative arm of PPP while enhancing the non-oxidative arm, thereby providing R5P essential for nucleotide synthesis and cellular proliferation. In addition, HIF-1 inhibits pyruvate entry into the mitochondrial TCA cycle by promoting the expression of pyruvate dehydrogenase kinase (PDK), an enzyme which inhibits PDH activity, and activates expression of lactate dehydrogenase A (LDH-A) which converts pyruvate to lactate. The latter reaction regenerates NAD^+^ necessary for maintaining oxidation of glucose by glycolysis (Brahimi-Horn and Pouyssegur, 2009; Semenza, 2009; Zhao et al., 2010).

Although HIF-1 inhibits flux through the TCA cycle, citrate production can be maintained in the presence of active HIF-1 through a reverse TCA reaction which generates isocitrate/citrate by reductive carboxylation from glutamine-derived α-ketoglutarate (Figure 4) catalyzed by isocitrate dehydrogenase 2 (IDH2) (Wise et al., 2011).

#### Myc

The oncogene Myc is another important transcriptional regulator of carbon and nitrogen metabolism. Myc is activated downstream of growth factor signaling pathways, in particular of those involving RAS (Morrison, 2012; Chesney and Telang, 2013). Myc enhances glutamine uptake and glutaminolysis by positively regulating the expression of glutamine transporters (e.g., SLC1A5) and glutaminase (GLS), thereby supplementing the mitochondrial TCA cycle by the generated α-ketoglutarate (see Figure 4B). In addition, Myc promotes biosynthesis of nucleotides and amino acids through increased production of the necessary TCA intermediates *via* enhanced glutaminolysis, but also by enhanced transcription of genes which encode enzymes involved in the biosynthesis of these compounds (Tong et al., 2009). Myc in collaboration with HIF-1 may also stimulate the conversion of glucose to lactate by activating the expression of the genes encoding GLUT1, HK2, LDH-A, and pyruvate kinase M2 (PKM2) (Yeung et al., 2008; Dang et al., 2009).

#### Pyruvate kinase (PK)

Pyruvate kinase (PK), which catalyzes the last step of glycolysis converting phosphoenolpyruvate (PEP) to pyruvate with concomitant ATP production, represents an important metabolic switch point. This enzyme exists in two isoforms, PKM1 and PKM2. The constitutively active PKM1 is the major PK controlling the glycolytic flow into the TCA cycle in non-proliferating cells. PKM2, a less active isoform, has been shown to be involved in cancer metabolism and tumor growth (Christofk et al., 2008). Myc enhances the expression of PKM2. Activity of PKM2 is allosterically activated by the upstream glycolytic intermediate fructose-1,2-diphosphate (FBP). Growth factor signaling leads to the release of FBP from PKM2 resulting in its decreased pyruvate kinase activity. This causes the accumulation of upstream glycolytic intermediates and activation of the PPP which provides R5P and NADPH. Thus, the switch to PKM2 favors anabolic processes, especially biosynthesis of serine/glycine, nucleotides and the antioxidant glutathione (Soga, 2013). PKM2 can also act as a protein kinase using PEP as a phosphate donor, thereby converting PEP to pyruvate without ATP generation (Gao et al., 2012). The switch of PKM2 between the protein kinase activity (in the dimer form) and pyruvate kinase activity (in the tetramer form) may be an important factor in cell proliferation (Gao et al., 2013).

#### p53

The p53 transcription factor, an extensively studied key tumor suppressor, is known to regulate in particular cell cycle arrest, apoptosis and DNA repair (Levine and Oren, 2009). But p53 is also an important regulator of carbon and energy metabolism. As a potent inhibitor of glycolysis (Vousden and Ryan, 2009), wild-type p53 downregulates glucose uptake and promotes expression of the tumor suppressor TP53-inducible glycolysis and apoptosis regulator (TIGAR). TIGAR is a phosphatase that lowers the level of fructose-2,6-diphosphate (F26BP), an allosteric activator of PFK1 (Figure 2). A reduced level of this metabolite therefore inhibits the glucose flux through glycolysis and may direct glucose-6P to PPP (Olovnikov et al., 2009). However, wild-type p53 binds directly to G6PDH thereby inhibiting its enzymatic activity which limits the glucose flux through PPP. Mitochondrial respiration is enhanced by p53-mediated induction of the cytochrome c oxidase assembly protein 2 (SCO2), an important regulator of cytochrome C oxidase. Another p53-activated metabolic target gene is GLS2 which encodes glutaminase 2. As described above, this enzyme converts glutamine to glutamate which—upon deamination to 2-oxoglutarate—replenishes the TCA cycle thereby enhancing the rate of the TCA pathway and OXPHOS.

Tumor-associated gain-of-function mutant p53 proteins have been identified (Brosh and Rotter, 2009) which may activate different metabolic pathways, e.g., the mevalonate pathway and hence lipid metabolism, glucose uptake with subsequent enhanced glycolysis or loss of the ability to block G6PDH activity, thereby activating the PPP (Jiang et al., 2011).

Metabolic effects mediated by p53 may also be due to its ability to inhibit the PI3K/Akt/mTOR pathway through the two tumor suppressors, phosphatase and tensin homolog (PTEN) and AMP-activated protein kinase (AMPK).

#### PTEN

PTEN is a phosphatidylinositol-3,4,5-triphosphate (PIP3) 3-phosphatase which preferentially dephosphorylates phosphoinositides. Through this activity, PTEN reduces the intracellular level of PIP3, thereby negatively affecting the PI3K/Akt signaling pathway (Song et al., 2012).

#### AMPK

AMPK is a key regulator of energy metabolism and glucose homeostasis. Liver kinase B1 (LKB1)-mediated phosphorylation activates AMPK by many stimuli, including cellular stress, hormones, cytokines and adipokines (Jones et al., 2005; Shackelford and Shaw, 2009; Hardie, 2011). AMPK inhibits mTORC1 and stimulates catabolic rather than anabolic pathways. Under low cellular energy conditions (high AMP/ATP ratio), AMPK triggers glycolysis if sufficient external glucose is provided. This results in decreased AMP and glucose levels. At low glucose level, sustained activity of AMPK activates p53 which—as described above—intervenes with glycolysis, PPP and OXPHOS thereby slowing down and balancing carbon flux through glycolysis and PPP (Jiang et al., 2014; Patra and Hay, 2014) and promoting OXPHOS. P53 further up-regulates transcription of the *SESN* gene and the produced sestrin proteins stimulate AMPK, thus eliciting a positive feedback loop.

#### RB

The retinoblastoma protein (RB) is a tumor suppressor protein that is absent or inactivated in several human cancers. The best-characterized function of RB is the prevention of excessive cell growth by inhibiting premature cell division. When the cell is ready to divide, RB is phosphorylated, becomes inactive and allows cell cycle progression. RB is involved in metabolism through the transcriptional regulation of enzymes essential for nucleotide biosynthesis (Angus et al., 2002) and seems to be involved in switching an oxidative to a glycolytic metabolism (Nicolay and Dyson, 2013).

Lastly, it should be noted that many of the metabolic regulators converge or depend for activation directly or indirectly on mTORC1, the central check point of cell proliferation (Dunlop and Tee, 2009) and show mutual interactions (Figure 5B). Furthermore, it is worth mentioning that the interaction of members of the NF-κB family of pleiotropic transcriptional factors with p53, HIF-1 and other metabolic regulators also influences the energy metabolism in mammalian cells (reviewed in Johnson and Perkins, 2012).

## Immune cells of the innate immune system (IIS) as foes and hosts for IBPs

In contrast to environmentally exposed interfaces, mammalian tissues are kept free of microorganisms by an efficient defense system including humoral and cellular components. Here, we will focus on the early phase of bacterial infections when the first line of defense, mainly comprising the IIS, is activated.

While commensal bacteria (as members of the healthy microbiota) support the host immune system by nutrient- and metabolite-dependent mechanisms (Brestoff and Artis, 2013), bacterial pathogens, especially IBPs, are able to break the cell and tissue sterility (Odegaard and Chawla, 2013) by using—under certain conditions—some of the immune cells, especially those of the IIS, as host cells where they may successfully survive, replicate and counteract the critical defense responses of these cells (Diacovich and Gorvel, 2010; Swart and Hensel, 2012).

The IIS consists of a collection of different subsystems (Medzhitov, 2007a). The most essential cellular players of the IIS are cells of the MPS, including MOs, MPs, DCs, and NPs as well as mucosal epithelial cells (MECs) and skin keratinocytes (SKs) (Guilliams et al., 2014). The specific molecules produced and in part secreted by these cell types upon activation act as signals for further immune responses or as direct antimicrobial agents. However, these cells may also serve as host cells for specific IBPs.

All cell types of the IIS and in particular MPs which show a high local and physiological flexibility (Murray and Wynn, 2011; Guilliams et al., 2014; Murray et al., 2014; Gross et al., 2015) express various sets of membrane-inserted, surface-exposed and cytosolic pattern recognition receptors (PRRs), which, as sensors for microbial pathogens, include the membrane-associated Toll-like receptors (TLRs) and C-type lectins, as well as the cytosolic nucleotide-binding oligomerization domain (NOD)-like receptors (NLRs) and the retinoic acid-inducible gene I (RIG.I)-like receptors (RLRs) (Kawai and Akira, 2011). The most important PRRs for recognition of bacterial pathogens are TLRs and NLRs. The major pathogen-associated molecular patterns (PAMPs^1^) recognized by TLRs include lipopolysaccharide (LPS), lipoteichoic acid (LTA), peptidoglycan (PGN), and other cell envelope components of Gram-positive and Gram-negative bacteria, flagellin, CpG DNA, RNA (Medzhitov, 2007a,b; Kawai and Akira, 2011; Oldenburg et al., 2012; Krüger et al., 2015), as well as cyclic di-AMP (Woodward et al., 2010; Commichau et al., 2015; Whiteley et al., 2015) and also the host-derived danger-associated molecular patterns (DAMPs) (Escamilla-Tilch et al., 2013) representing endogenous molecules released from damaged host cells including mitochondrial DNA (Mills et al., 2017),. The cytosolic NLRs comprise more than 20 members (Franchi et al., 2008). Among the NLRs, NOD1 and NOD2 recognize specific fragments of the bacterial PGN, but for most NLRs the specific interacting ligands are yet unknown.

Exposure of MAMPs to PRRs differs between intracellular and extracellular bacterial pathogens. Extracellular bacterial pathogens may release MAMPs through various processes which then can directly bind to PRRs on immune cells, triggering host responses. In contrast, IBPs produce MAMPs which—due to their intracellular localization—may have more limited access to the immune system. The host responds to these MAMPs likely through different programs; it becomes increasingly clear that exosomes and other extracellular vesicles released from infected cells play an important role in this dissemination process (for a recent review, see Schorey et al., 2015). These vesicles not only include MAMPs, but also T- and B-cell antigens, as well as pathogen-derived virulence factors.

Interactions of MAMPs with TLRs and with NOD1/2 activate the MyD88-dependent signaling pathway leading (among others) to the activation of the transcription factors NF-kB, AP-1 and interferon regulatory factors (IRFs). These transcription factors are known to induce the expression of genes encoding the production of pro-inflammatory cytokines and chemokines, pro-IL-1ß, pro-IL18 and IL-8, TNF-α and of type I interferons (especially IFN-α and IFN-ß) essential for further immune responses (Ozinsky et al., 2000).

A subset of the NLRs (AIM2, IPAF, NLRP1, and NLRP3) initiates, e.g., by interaction with bacterial flagellin and DNA, the formation of inflammasomes, multiprotein complexes present in epithelial cells and immune cells of the myeloid lineage. The inflammasomes activate in particular caspase 1 and 11 which cleave pro-IL-1ß and pro-IL-18 to their active forms which are subsequently released, followed by pyroptosis. These cytokines in their mature form are key regulators of the host response against microbial pathogens (Schroder and Tschopp, 2010).

A hallmark of activated professional phagocytes (MOs, MPs, and NPs) and activated MECs is the production of microbicidal ROS and reactive nitrogen intermediates (RNIs) catalyzed by the NADPH oxidase and the inducible NO synthase (iNOS), respectively. In addition, the proinflammatory cytokines IL-1ß, IL-6, and TNF-α cause an inflammatory environment. Finally, restriction of accessible intracellular iron ions, essential for IBP replication, appears to be also an important defense mechanism of MPs and possibly other immune cells (Nairz et al., 2014, 2015).

PMNs represent the most abundant phagocytic cell population in human blood. These professional phagocytes are rapidly mobilized to sites of infection (Kennedy and Deleo, 2009) and, upon phagocytosis, kill bacterial pathogens and other microbes by a combination of ROS, cytotoxic granule components, hydrolytic enzymes and antimicrobial peptides which generate a highly lethal intraphagosomal environment (Nauseef, 2007; Kennedy and Deleo, 2009). NPs are short-lived and are pre-programmed to undergo under normal conditions spontaneous apoptosis, 18–24 h after release into circulation.

Besides these professional phagocytes, invading microorganisms encounter less aggressive IIS immune cells which form a dendritic network in lymphoid and non-lymphoid tissue (LT and NLT) and therefore are named DCs. The DCs comprise several subtypes with characteristic properties, such as sentinel functions and transport antigen and microorganisms to lymph nodes for the induction of an adaptive immune response (Mildner and Jung, 2014).

## Metabolic shifts in immune cells of the IIS triggered by activating or inhibiting mediators (“immunometabolism”)

The past decade has experienced a focus on the convergence of metabolism and immune functions for which the term “immunometabolism” has been coined (for reviews, see Rathmell, 2012; Pearce and Pearce, 2013; Delgoffe and Powell, 2015; O'Neill and Pearce, 2016). Like most terminally differentiated mammalian cells, the non-activated immune cells of the IIS perform a quiescent metabolism which is characterized by a low glucose flow through glycolysis and the TCA cycle and the formation of ATP predominantly by OXPHOS often fueled by ß-oxidation of fatty acids/lipids in the mitochondria (DeBerardinis et al., 2006). Their biosynthetic activities are low under these conditions and even in presence of sufficient nutrient supply, trophic signals, such as cytokines must be present to avoid cell death (Frauwirth and Thompson, 2004). This quiescent metabolic state is activated in all immune cells of the IIS by various ligand/receptor interactions as shortly summarized in the following.

### Neutrophils, dendritic cells, and macrophages

Resting **NPs** which contain few mitochondria and consume little oxygen (Van Raam et al., 2008) are mainly activated by TLR agonists, formylated peptides or by the local growth factor G-CSF. Activated NPs show increased oxygen consumption, glucose uptake, and now produce ATP exclusively by aerobic glycolysis (Gabig et al., 1979). PPP activity required especially for the generation of NADPH is also induced. NADPH is essential for the induction of NADPH oxidase responsible for the production of H_2_O_2_, the characteristic microbicidal product of NPs. Netosis, the special ability of NPs to release extracellular nets containing DNA and microbicidal proteins (Urban et al., 2009) is also dependent on NADPH oxidase (Kirchner et al., 2012).

**DCs**, a heterogeneous cell population, reside in tissues exposed to the external environment and represent a surveillance system sensing infections and the first line of defense against invading pathogens (Dominguez and Ardavin, 2010; Arnold-Schrauf et al., 2014; Mildner and Jung, 2014; Durai and Murphy, 2016). DCs also act at the transition from IIS and AIS by presenting microbial antigens to T cells and providing inflammatory signals that modulate T cell differentiation (Steinman, 2012).

Resident DCs undergo, upon interaction of microbial MAMPs and cellular DAMPs with the corresponding PRRs, a rapid switch from the resting metabolic state (characterized by fatty acid oxidation and OXPHOS) to an activated state. This is accompanied by the extensively studied induction of cytokines, chemokines and other factors functioning in IIS (and AIS). Most of the encoding genes are controlled by NF-κB, the expression of which is also highly induced under these conditions. The activated metabolic state of (MO-derived) DCs (triggered e.g., by *E. coli* or only by its LPS) is characterized by the induced expression of genes encoding proteins involved in glucose catabolism via glycolysis and PPP and transcription factors activating these pathways, in particular HIF-1 (Huang et al., 2001; Jantsch et al., 2008). An in-depth analysis (Krawczyk et al., 2010) showed that the interactions of TLR2, TLR4, and TLR9 with their corresponding ligands, i.e., bacterial PGN, LPS, and CpG, respectively, cause a switch from the OXPHOS-engaged resting state to the activated state characterized by aerobic glycolysis and lactate production similar to the “Warburg effect” in cancer cells (Warburg et al., 1927). These so-called Tip-DCs^2^ induce expression of iNOS resulting in increased NO production from arginine which seems to be responsible for the breakdown of mitochondrial respiration (Everts et al., 2012). This TLR-induced metabolic change is driven by the PI3K/Akt pathway (probably in a MyD 88-dependent manner (Gelman et al., 2006) and relies on HIF-1α (Jantsch et al., 2008). AMPK and the anti-inflammatory IL-10 antagonize the TLR-induced activation process (Krawczyk et al., 2010; Carroll et al., 2013). In contrast to cancer cells and activated T and B cells in which the metabolic switch to aerobic glycolysis causes enhanced cell division, the Warburg effect in DCs is mainly associated with increased biosynthetic activities (in particular production of immune mediators, like IL-12, IL-6, and TNF) rather than with cell proliferation. A more detailed overview on the present knowledge of the metabolic changes in the various activated DC subtypes is given in a recent review (Pearce and Everts, 2015).

**MPs** can be functionally grouped, in a simplified view, into pro-inflammatory bactericidal M1 and anti-inflammatory M2 subsets (Mantovani et al., 2005; Mosser and Edwards, 2008). *In vivo* studies show, however, that a broad spectrum of MP subtypes seems to exist with considerable plasticity rather than solely the M1- or M2-polarized subtypes (Murray and Wynn, 2011; Stewart et al., 2012; Vergadi et al., 2017).

Classical activation of resident, metabolically quiescent MPs, e.g., by LPS and IFN-γ, induces the M1 phenotype which is characterized (similar to the activated DC state) by HIF-1 activation, leading to increased glucose uptake and enhanced aerobic glycolysis, increased flux through the PPP, inhibition of mitochondrial OXPHOS and enhanced lipid biosynthesis (Rathmell, 2012). The LPS/TLR4-mediated activation also results in increased RNI and ROS production which mainly contributes to the antimicrobial activity of M1 macrophages. ROS is not only produced by the induced phagosomal NADPH oxidase, but also in the mitochondria by translocation of the tumor necrosis factor receptor-associated factor 6 (TRAF6) into these organelles (West et al., 2011). These MPs use significant amounts of the glycolytically generated ATP to maintain the mitochondrial membrane potential, thereby preventing apoptosis (Garedew et al., 2010). The M1 state is characterized by the production of pro-inflammatory cytokines TNF-α, IL-1ß, and IL-12.

Alternatively activated M2 macrophages (activated by IL-4 and IL-13) exhibit AMPK-controlled enhanced mitochondrial OXPHOS, fatty acid oxidation and a low rate of glycolysis (Vats et al., 2006). These MPs produce the anti-inflammatory cytokines IL-4 and IL-5. IL-4 induces the expression of arginase 1, a characteristic marker of M2 macrophages. This enzyme converts L-arginine to L-ornithine (and urea) which may lead via glutamic semialdehyde to L-proline. In contrast, in M1 macrophages imported L-arginine is used for the generation of NO catalyzed by iNOS (Gordon and Martinez, 2010; Odegaard and Chawla, 2011; Martinez and Gordon, 2014).

An interesting finding concerning the M1/M2 polarization has been reported by Haschemi et al. (2012). These authors showed that the sedoheptulose kinase SHPK (formerly carbohydrate kinase-like CARKL), which catalyzes an orphan reaction in the PPP, is an important modulator for M1/M2-polarization during LPS activation. SHPK downregulation (or loss) induced by LPS leads to induction of M1-like polarization while SHPK upregulation results in M2-like activation of MPs.

In summary, the metabolic hallmarks of activated NPs, DCs, and M1 macrophages are aerobic glycolysis, absence of OXPHOS and enhanced PPP leading to increased biosynthetic activity but not to cell proliferation. The high rate of glycolysis seems to be necessary to meet the increased demand of energy and precursors for the biosynthesis of antimicrobial metabolites and pro-inflammatory proteins. In contrast, M2 macrophages perform OXPHOS, fuelled mainly by fatty acid oxidation, while aerobic glycolysis does not occur.

### Mucosal epithelial cells and keratinocytes

Surface epithelia, such as the mucosal epithelia and the skin, are only one or a few cell layers thick but represent effective protective barriers against most microorganisms. Both, MECs, and SKs are non-professional phagocytes, but produce a diversity of antimicrobial proteins that directly kill or inhibit the growth of most of the frequently encountered microorganisms. Due to their antimicrobial potential (Gallo and Hooper, 2012), MECs and SKs are also considered as part of the IIS. Many IBPs are able to actively invade the epithelial cells and to replicate in the intracellular environment during infection (Pizarro-Cerdá and Cossart, 2006). The metabolic responses of MECs and SKs caused by IBP infections are poorly studied.

In response to disturbance of the tissue integrity, due to different events including infections by IBPs, MOs and NPs are recruited to the sites of infection where the activated phagocytes contribute to local inflammation. These inflammatory sites are characterized by low concentrations of oxygen and glucose, but high levels of lactate and ROS (Saadi et al., 2002; Campbell et al., 2014). The surrounding epithelial cells are affected by the oxygen depletion and ROS generation leading (among others) to HIF-1 stabilization (Campbell and Colgan, 2015) and enhanced HIF-regulated expression of antimicrobial factors (Peyssonnaux et al., 2008) and probably also to HIF-triggered shifts of metabolic pathways (Kominsky et al., 2010).

## Metabolic adaptations of IIS cells and the IBPs

The responses of the effector immune cells to microbial pathogens, especially to IBPs, are aimed to eliminate (through the various induced defense responses discussed above) the respective pathogen and to return to cell sterility (Odegaard and Chawla, 2013). On the other hand, as repeatedly mentioned, effector immune cells, in particular those of the IIS, may also serve as niches for intracellular survival, replication and dissemination of many IBPs (Kumar and Valdivia, 2009; Rikihisa, 2010; Fabrik et al., 2013; Fredlund and Enninga, 2014). The fact that the same cell type is able to perform these two apparently contradictory functions and may allow intracellular replication of IBPs with highly diverse metabolic potentials suggests that different metabolic adaptation processes have to take place in both partners. Several possible solutions to this “paradox problem” are outlined in **Figure 7**. In the following, we will discuss investigations that shed some light on this intriguing problem.

### How do IBPs adapt to the metabolic conditions inside immune cells?

Different IBPs prefer different carbon compounds for energy generation and production of necessary catabolic intermediates (Eisenreich et al., 2010; Abu Kwaik and Bumann, 2013; Barel et al., 2015). The few metabolic generalists among the IBPs, mainly the facultative intracellular *Enterobacteriaceae* (*Salmonella, Shigella* and the related enteroinvasive *E. coli*) and possibly also *Brucella* spp. seem to use glucose as preferred carbon source for their intracellular catabolic and anabolic activities (Bowden et al., 2009; Götz et al., 2010; Xavier et al., 2013; Waligora et al., 2014). However, glucose is also the primary carbon source for metabolically activated immune cells that serve as host cells for IBPs. Extensive glucose deprivation by the IBPs may therefore lead to cell death by apoptosis or pyroptosis (Fink and Cookson, 2007; Suzuki et al., 2007; Lembo-Fazio et al., 2011).

**Figure 6:**
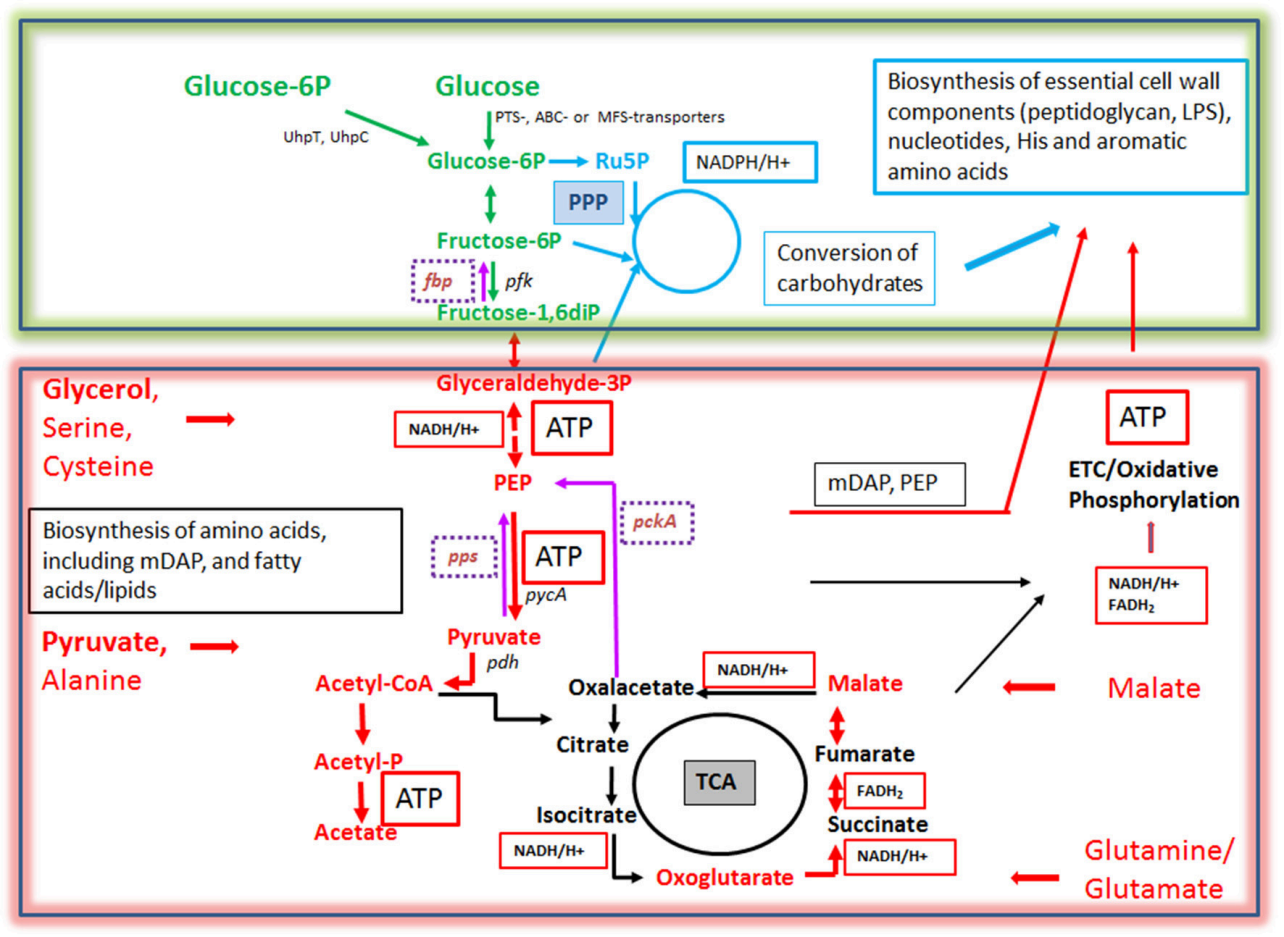
Bipartite metabolism of IBPs aiming to avoid the exclusive use of glucose (an indispensable carbon source of their mammalian host cells) by the IBPs for their intracellular metabolism. This strategy divides the bacterial carbon metabolism into two modules. Module 1 (green-framed), contains the upper (energy-dependent) part of the glycolytic pathway, as well as the oxidative and non-oxidative arms of PPP. These metabolic pathways are fed mainly by glucose, glucose-6P or polymers (e.g., glycogen) yielding these hexoses and deliver intermediates essential for the (energy-dependent) biosynthesis of cell envelope components, nucleotides, aromatic amino acids and NADPH, a co-factor essential for several biosynthetic pathways (especially fatty acids/lipids). The synthesis of certain cell envelope components and NADPH is indispensable for intracellular growth of the IBPs while all other components, including aromatic amino acids and nucleotides produced in module 1 can be also provided by the host cell (see Figure 3). Module 2 (red-framed) comprises the lower part of glycolysis, the TCA cycle and electron transport chain (ETC). Module 2 can be fed by C_3_-carbon sources, such as glycerol-(3P), serine, cysteine and pyruvate, by glutamine/glutamate and possibly by TCA intermediates, e.g., malate. It delivers mainly ATP by (a) substrate phosphorylation through NAD-dependent oxidation of these carbon sources and (b) by oxidative phosphorylation *via* ETC which converts NADH back to NAD necessary for maintaining the oxidation of the above carbon substrates. Module 2 delivers also intermediates for the biosynthesis of several amino acids, all of which can be imported from the host cell. Indispensable is, however the production of meso-diaminopimelic acid (mDAP) and PEP for the synthesis of peptidoglycan and of Ac-CoA for the biosynthesis for specific cell-envelope-associated fatty acids/lipids (see Figure 3).

On the other hand, IBPs that have adapted to a mainly intracellular life, like *Rickettsia, Chlamydia, Coxiella, Francisella*, and *Legionella* are more or less auxotrophic and often lack pathways and reactions for efficient glucose uptake and/or catabolism. These IBPs, but even the above mentioned metabolic generalists may pursue an intracellular metabolic strategy to achieve optimal intracellular replication which we recently termed “bipartite metabolism” (Grubmüller et al., 2014; Abu Kwaik and Bumann, 2015; Eisenreich et al., 2015). In this scenario, a combination of several host cell-derived carbon compounds drives the intracellular microbial metabolism and thereby allows a fine-tuned adaptation to the host cell (Figure 6). One (or more) host cell-derived energy-rich carbon substrate(s), less critical for the host cell metabolism than glucose, may function as major energy source for ATP production by substrate phosphorylation or oxidative phosphorylation *via* the ETC. These substrates include glycolysis-derived compounds, like glycerol(-3P), pyruvate and lactate, but also TCA intermediates, like succinate and malate, or amino acids that can be converted to such substrates, such as serine, alanine, aspartate, glutamate, and fatty acids.

More specifically, for this purpose *L. monocytogenes* seems to use glycerol (Eylert et al., 2008), *Shigella* pyruvate (Kentner et al., 2014), *Francisella tularensis* various amino acids, glycerol, glycerol-3P, or pyruvate (Brissac et al., 2015), *Salmonella enterica* glycerol, fatty acids and lactate (Steeb et al., 2013), *Legionella pneumophila* amino acids especially serine (Price et al., 2011; Schunder et al., 2014), *Brucella abortus* possibly glutamate (Zuniga-Ripa et al., 2014), *Chlamydia trachomatis* possibly malate (Iliffe-Lee and Mcclarty, 2000; Mehlitz et al., 2017), and *Mycobacterium tuberculosis* fatty acids, cholesterol (Ehrt et al., 2015; Lovewell et al., 2016) or glycolytic C_3_ compounds (Beste et al., 2013), respectively. These carbon substrates are not necessarily utilized for the generation of hexoses (and subsequently other carbohydrates needed for cell envelope synthesis) *via* gluconeogenesis. Indeed, several IBPs (*Rickettsia, Chlamydia, Coxiella*, and *Legionella*) lack the typical bacterial type I-III fructose-1,6-diphosphatases (FBPase, an essential enzyme for gluconeogenesis). It is, however, possible that these IBPs, some of which (e.g., *L. pneumophila*) are definitely able to perform gluconeogenesis, might compensate the missing conventional FBPase by alternative FBPase activities, e.g., by the reversible pyrophosphate-dependent phosphofructokinase or fructose-6-phosphate aldolase (Schürmann and Sprenger, 2001; Arimoto et al., 2002; Ganapathy et al., 2015). On the other hand, in *Brucella abortus* which possesses type I and type II FBPases, both enzymes are dispensible for intracellular replication (Zuniga-Ripa et al., 2014), whereas in *Francisella tularensis* inactivation of the *glpX* gene (encoding a type II FBPase) leads to reduced intracellular multiplication (Brissac et al., 2015), indicating the need of gluconeogenesis for intracellular replication of this IBP particularly in those stages of infection in which glucose is limited. However, when glucose is available most IBPs take up glucose in addition to the above mentioned carbon source(s) from the host cell, but use it for anabolic rather than for energy-producing catabolic pathways (Figure 6). In this context, it is interesting to note that most IBPs (exceptions are the facultative intracellular *Enterobacteriaceae*) lack the highly efficient glucose-specific phosphoenolpyruvate-dependent transport system (PTS), but may possess less efficient ABC or major facilitator superfamily GLUTs (Aguilar et al., 2012). Interestingly, *L. monocytogenes* possess a large number of PTS, but lack the most efficient glucose-specific PTS-G (Stoll and Goebel, 2010). Several IBPs are able to import phosphorylated hexoses (especially glucose-1P and/or glucose-6P, deriving eventually from host cell glycogen) by means of specific transporters: UhpT in case of *L. monocytogenes* (Chico-Calero et al., 2002) and UhpC in case of *Chlamydia pneumoniae* (Schwöppe et al., 2002) and *Legionella pneumophila* (Schwöppe et al., 2002; Chien et al., 2004). The expression of these transporters may be induced under the intracellular conditions (Joseph et al., 2006).

The imported glucose or glucose-P seems to be mainly channeled into the PPP. NADPH, produced in the oxidative branch of the PPP (present in IBPs with the exception of *Francisella, Coxiella*, and *Rickettsia* spp.), serves as cofactor for the reductive biosynthesis of nucleotides and fatty acids and for the generation of glutathione which provides protection against oxidative damage. Through the non-oxidative arm of the PPP (present in all IBPs except *Rickettsia* spp.) specific phosphorylated carbohydrates are generated which are required for the biosynthesis of aromatic amino acids, nucleotides and bacterial cell envelope components.

Most other basic anabolic compounds, especially amino acids (even those which in principle could be synthesized by the IBPs), are imported from the host cell (Grubmüller et al., 2014). An exception is meso-diaminopimelate (mDAP), an essential component of peptidoglycan synthesis which cannot be delivered by the host cell and has to be apparently produced by the IBPs (Figure 1). Among all IBPs for which genome sequences are known, only *Francisella tularensis* and other *Francisella* spp. lack most genes encoding the enzymes necessary for mDAP biosynthesis.

The withdrawal of amino acids from the host cells by the IBPs will cause amino acid starvation in the host cells (Tattoli et al., 2012; Tsalikis et al., 2013) which may lead, as discussed below, to induction of “prosurvival autophagy” thus prohibiting early host cell death by apoptosis/pyroptosis. Some IBPs possess anti-autophagic properties and in this case the induced autophagy of the host cell may even support intracellular replication of the IBPs (see below).

The concept of bipartite metabolism may also explain the frequently observed persistent state of IBPs, i.e., their intracellular survival without active replication. Interruption of the major glucose (or glucose-6P) supply to the IBP will stop bacterial cell wall synthesis and cell division, but as long as the catabolism of an alternative carbon substrate provides sufficient ATP (and eventually necessary amounts of phosphorylated carbohydrates generated by gluconeogenesis and PPP) to maintain the integrity of indispensable macromolecules (especially of DNA and cell envelope structures), the IBP will survive but not multiply.

### Antimicrobial responses of host cells and the bacterial countermeasures may require alterations of the host cell metabolism

As outlined above, the well-characterized defense mechanisms executed by the immune cells of the IIS include ROS and RNI production, synthesis of antibacterial peptides, induction of inflammasomes with the concomitant synthesis of inflammatory cytokines (IL1-ß and IL18) and subsequent pyroptosis as well as induction of autophagy (Sauer et al., 2010). These defense mechanisms are predominantly triggered by the interactions of different bacterial MAMPs with their corresponding TLRs and NLRs. Certain T3SS- and T4SS-effector proteins, e.g., SopE and SopB of *S. Typhimurium* secreted by T3SS-1, can also promote inflammatory host defense responses (Bruno et al., 2009).

The induced expression of these antimicrobial host responses requires activation of the metabolism of the immune cells involved (Lewis et al., 2011; Amiel et al., 2012; Everts et al., 2012). Interactions of LPS with TLR4 and of LTAs and lipoproteins with TLR2 and TLR6—presumably early events in the encounter of Gram-negative and Gram-positive IBPs, respectively with the IIS immune cells *in vivo*—trigger these defense reactions and activate the metabolism of the initially metabolically quiescent phagocytes (Krawczyk et al., 2010). This activation step leads to PI3K/Akt-mediated induction of aerobic glycolysis and NF-kB activation causing (among others) increased production of ROS and NO with subsequent loss of OXPHOS and inhibition of the TCA cycle (Everts et al., 2012). Induced expression of PKM2 seems to play an important role in this metabolic switch and is characteristic to inflammatory immune cells, such as M1 macrophages, Tip-DCs (M1-related cells that release TNF and NO, see above) (Schmid et al., 2012; Guilliams et al., 2014; Martinez and Gordon, 2014; Murray et al., 2014) and Th17 cells (Korn et al., 2009). This type of inflammatory cell metabolism represents, however, a rather hostile setting for the intracellular replication of invading IBPs (Price and Vance, 2014; Palsson-Mcdermott et al., 2015). Indeed, mice with impaired NO generation show dramatically enhanced bacterial proliferation in Kupffer cells upon infection with *S. Typhimurium* (Vazquez-Torres et al., 2004), suggesting that, without the antimicrobial NO production, intracellular replication of the *S. Typhimurium* in these metabolically activated MPs proceeds much more efficient.

To counteract the plethora of antimicrobial host cell reactions, IBPs have developed a variety of defense mechanisms: (a) inhibition of MAMP/PRR recognition through modification and shutting down the production of MAMPs, like LPS, LTA, and flagellin, (b) inactivation of ROS by induced production of superoxide dismutase and catalase, (c) reduction of the NO level through induction of arginase, (d) inhibition of inflammasome formation and pyroptosis, and (e) suppression of autophagy (Hedrick, 2004; Shen and Higgins, 2006; Bhavsar et al., 2007; Coats et al., 2007; Phalipon and Sansonetti, 2007; Ishii et al., 2008; Pieters, 2008; Taxman et al., 2010; Kullas et al., 2012; Fabrik et al., 2013; Smith and May, 2013; Al-Khodor et al., 2014; Okumura and Nizet, 2014; Wynosky-Dolfi et al., 2014). The realization of most of these bacterial countermeasures against the host cell defense reactions requires, however, a host cell metabolism that allows active intracellular replication of the IBPs and it remains to identify the appropriate metabolic host cell conditions that activate the IBP-specific countermeasures.

### Metabolic activation of quiescent host cells that may prohibit or favor intracellular growth of IBPs

The paradox situation that the same immune cells may carry out antimicrobial (i.e., kill IBPs) but also promicrobial functions (i.e., serve as convenient host cells for the IBPs) was discussed by the example of MPs in a recent review (Price and Vance, 2014). An important message was that the anti-inflammatory M2 but not the inflammatory M1 phenotype of activated MPs represents a comfortable replication niche for IBPs. Indeed, recent studies (Eisele et al., 2013; Xavier et al., 2013) showed that alternatively activated M2 macrophages represent the predominant MP population inhabited by replicating *Salmonella* and *Brucella* strains, respectively, during chronic infection in the mouse model. This ability correlates with higher levels of unconsumed glucose in the M2 compared to M1 macrophages. Signal transduction through the nuclear peroxisome proliferator-activated receptors (PPAR-γ and PPAR-δ), respectively, was shown to be crucial for this process. Induction of these metabolic regulators enhance OXPHOS and fatty acid ß-oxidation in M2 macrophages thereby leading to increased levels of unconsumed glucose. M1 macrophages on the other hand catabolize glucose through the highly activated glycolytic pathway and thereby withdraw the bacteria this essential carbon source.

These findings led to the suggestion that the PPAR-induced increase in intracellular glucose availability may be a rather general mechanism promoting growth of persistent pathogens within M2 macrophages (Xavier et al., 2013). Indeed, M2 polarization of human macrophages was recently shown to favor also replication of *Chlamydia pneumoniae* (Buchacher et al., 2015).

However, the typical M2 metabolism does not seem to be the only metabolic condition which supports replication and proliferation of IBPs. Established MO- and MP-like cell lines, such as J774A.1, P388.D1, RAW264.7, THP-1, and U-973, which are routinely used as host cells for studying intracellular replication of IBPs, perform a permanently activated metabolism which is mainly triggered by the constitutive expression of oncogenes (e.g., Myc), or by the inactivation of tumor suppressors (e.g., p53). In these cell lines, most IBPs are able to replicate efficiently, indicating that this type of activated, glycolysis-driven host cell metabolism also supports successful intracellular replication of many IBPs (Fuchs et al., 2012; Eisenreich et al., 2013; Kentner et al., 2014). The dysregulated metabolism of these transformed cell lines is clearly different from the metabolism of M2 macrophages (characterized by ß-oxidation of fatty-acids driven OXPHOS), but apparently provides all nutrients necessary for the bipartite metabolism of most IBPs.

It should also be noted that in contrast to *Salmonella* and *Brucella*, the intracellular replication capacity of *Listeria, Franciscella* and *Mycobacterium* strains is not influenced by the modulation of the PPAR levels and hence the intracellular glucose concentration of the host cells (Eisele et al., 2013).

**Figure 7:**
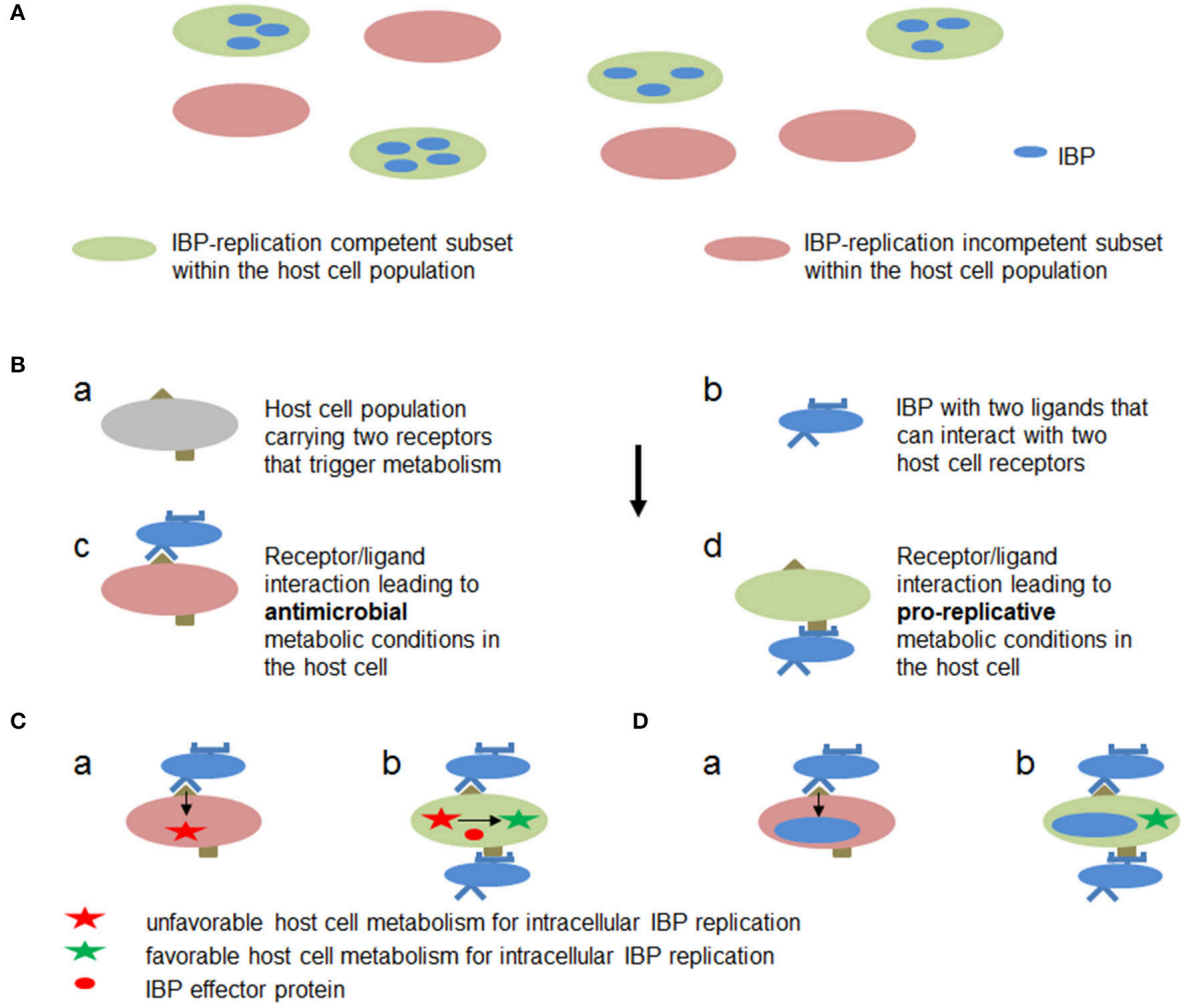
Hypothetic metabolic host cell conditions and interactions between host cell targets and IBP components leading to metabolic conditions in the host cell that are favorable or adverse for intracellular replication of IBPs. **(A)** Within a host cell population (e.g., MPs) metabolically different subsets (a and b) may already exist which allow (blue) or inhibit (yellow) active metabolism and hence replication of the IBP. **(B)** Cells within a host cell population may carry two receptors (a) which can interact with two different ligands of the IBP (b). One receptor/ligand combination (c) leads to an antibacterial metabolic host cell state (red), while the other (d) triggers metabolic host cell conditions that allow intracellular replication of IBP (green). **(C)** IBP (yellow circle) is internalized by the interaction of a host cell receptor (blue half circle) that triggers phagocytosis of the IBP but induces at the same time a host cell metabolism (a) that is unfavorable for intracellular IBP replication (red star). However, before or during internalization (b) the IBP may translocate an effector protein (red circle) via specific secretion (injection) systems that alters the host cell metabolism to become favorable for IBP replication (green star). **(D)** The IBP activates by a first interaction (a) a host cell metabolism with bacteriocidal activity. The IBP may inactivate, however, by a second bacterial ligand/host cell receptor interaction (b) the bacteriocidal activity thereby rendering the host cell metabolism favorable for IBP replication (green star).

These data show that not only the strategy exemplified by M1/M2-polarized MPs (strategy A, outlined in Figure 7A), but also other (still rather hypothetical) strategies (exemplified e.g., by Figures 7B–D) may result in a host cell metabolism that supports IBP replication: (A) in an already activated immune cell population different subsets may exist (e.g., M1/M2 macrophages and their subgroups Martinez and Gordon, 2014) that are either metabolically favorable or adverse for intracellular replication of IBPs, (B) anti- or pro-replicative metabolic conditions may be induced in individual cells of a quiescent immune cell population depending on the interaction between different host cell receptors and different IBP ligands, (C) before or shortly after internalization the IBPs may translocate effector protein(s) that specifically counteract the antimicrobial activities (e.g., ROS or RNI production) without changing the metabolic conditions of the host cell that are otherwise favorable for IBP replication, or (D) phagocytosis of the IBPs and activation of pro-replicative metabolic conditions in the immune cells may be separately triggered by interaction of different host cell receptors with different IBP-specific ligands.

### Interactions between host cell targets and bacterial effectors leading to metabolic changes within host cells that may support intracellular replication of IBPs

The data discussed in Interactions between host cell targets and bacterial effectors leading to metabolic changes within host cells that may support intracellular replication of IBPs, suggest that the exclusive use of glucose is not necessarily the best strategy for fueling the intracellular metabolism of all IBPs. Rather, IBPs according to their different metabolic capacities may depend—for performing an optimally balanced intracellular metabolism and hence efficient proliferation—on IBP-specific combinations of catabolic and anabolic metabolites that are provided by the host cell. This in turn requires IBP-specific metabolic conditions within (non-cancer) host cells which (*in vivo*) may already exist in a subset of a potential host cell population or may be induced by invading pathogens in metabolically quiescent (primary) cells through the interaction of specific bacterial ligands (e.g., effector proteins or other virulence factors) and appropriate host cell receptors (Figure 7).

As discussed in Metabolic activation of quiescent host cells that may prohibit or favor intracellular growth of IBPs, the alternatively activated M2 macrophages seem to represent such a subpopulation performing a metabolism that supports intracellular replication of certain IBPs (Price and Vance, 2014). Alternatively, specific T3SS- or T4SS-effector proteins or other virulence factors of IBPs which directly or indirectly interact with the above described central regulators of host cell metabolism, including PI3K, Akt, mTOR, cMyc, HIF-1α, PKM2, P53, TIGAR, PTEN, and AMPK, might also be candidates for modulating the metabolism of (quiescent) primary host cells in such a way that it becomes supportive for intracellular IBP replication. Indeed, interactions of IBP effectors with several of these oncogenes and tumor suppressors have already been demonstrated (Table 1). However, the outcome of these interactions has been so far mainly studied with respect to the pathogen internalization and/or immune responses, while the likely impact on the host cell metabolism has hardly been addressed. To the best of our knowledge, only few reports (see below) show how IBPs may manipulate the host cell metabolism, in particular that of immune cells by such interactions.

Gillmaier et al. (2012) showed that primary murine bone marrow-derived MPs (BMDMs) which perform in the uninfected state the typical quiescent OXPHOS-based metabolism (see above), are metabolically highly activated upon internalization of wild-type *L. monocytogenes*. However, in contrast to LPS- and IFN-γ-mediated metabolic activation, the *L. monocytogenes*-induced BMDM metabolism does not only lead to induction of the glycolytic pathway but also to induction of the initial steps of the TCA cycle thus enabling synthesis of citrate, its subsequent export into the cytosol and the generation of acetyl-CoA and oxaloacetate by ACL. This may allow enhanced anabolic activities in the BMDMs (especially fatty acid/lipid and amino acid biosynthesis). This type of induced host cell metabolism is similar to that occurring in the murine macrophage cell line J774 (its metabolism is permanently activated due to constitutively expressed Myc) where *L. monocytogenes* replicates without significantly altering the metabolism of these host cells. The efficiency and the mode of intracellular metabolism of *L. monocytogenes* are similar in the activated BMDM and in J774 cells (Gillmaier et al., 2012).

Interestingly, only a small population of the BMDMs (<5%) contains a large number (>20 bacteria/host cell) of replicating listeriae, suggesting that this type of metabolic activation, obviously favorable for intracellular replication of *L. monocytogenes*, may be induced only in a subset of the BMDMs. Notably, this *L. monocytogenes*-triggered metabolism of the BMDMs mimics more the growth factor-induced metabolism governed by the PI3K/Akt/mTORC1 pathway which leads to the activation of glycolysis, PPP and TCA (Kaplan et al., 2000; Jones and Thompson, 2009; Salminen and Kaarniranta, 2010). It has been shown that internalin B (InlB) of *L. monocytogenes* has structural and functional similarities to the hepatocyte growth factor (HGF), recognizes the same receptor Met as HGF and InlB/Met interaction activates PI3K (Ireton et al., 1996; Shen et al., 2000; Bierne and Cossart, 2002; Li et al., 2005; Niemann, 2013; Gessain et al., 2015). It is therefore intriguing to speculate that InlB/Met interaction in a subset of the BMDM population (Galimi et al., 2001; Moransard et al., 2010) might be responsible for the induction of the above described metabolism that supports intracellular *L. monocytogenes* replication. Direct experimental evidence for this hypothesis is still missing, but it has been shown (Gillmaier et al., 2012) that a non-pathogenic *prfA* mutant (also unable to express *inlB*) and heat-inactivated *L. monocytogenes* wild-type bacteria are unable to induce this type of metabolism in BMDM.

As discussed above, the tumor suppressor p53 blocks glucose uptake, glycolysis and PPP (Vousden and Ryan, 2009) whereas inactivation or down-regulation of p53 leads to the activation of these metabolic pathways which occurs in many cancer cells. Caco-2 cells contain a deleted and mutated p53 gene and no detectable p53 protein (Djelloul et al., 1997). These metabolically activated cells are excellent hosts for many IBPs including *L. monocytogenes*. Indeed, down-regulation of p53 in RAW264.7 and HeLa cells and p53 knockout mice also results in significantly increased intracellular replication of *L. monocytogenes*, whereas intracellular replication is inhibited when p53 was overexpressed in the two cell lines (Wang et al., 2016).

*Shigella flexneri* (which similar to *L. monocytogenes* replicates in host cells cytosol) may invade and multiply within MPs but rapidly induce pyroptotic cell death of these host cells (Suzuki et al., 2007). Epithelial cells of the intestine are the preferred host cells which allow efficient replication of *Shigella* upon oral infection. In the infected cells, degradation of p53 has been reported (Bergounioux et al., 2012) which is mainly caused by calpain protease activation, promoted by the T3SS effector protein VirA. The p53 degradation could activate the host cell metabolism, but the calpain activation causes also cell death and thus elimination of this replication niche (Bergounioux et al., 2012). However, IpgD, another T3SS effector of *S. flexneri*, dephosphorylates PIP2 and the resulting phosphatidylinositol-5-phosphate leads to the activation of the PI3K pathway which promotes phosphorylation of Akt (Pendaries et al., 2006). This Akt activation could also activate the host cell metabolism.

Down-regulation of p53 has also been demonstrated in human cells upon infection by different *Chlamydia* species (González et al., 2014; Siegl et al., 2014) and this down-regulation appears to be crucial for the efficient intracellular replication of *C. trachomatis* which in this case mainly occurs in a membrane-bound compartment, the inclusion. However, it is still unknown which chlamydial factor(s) is responsible for the p53 down-regulation and what are the metabolic consequences.

*Mycobacterium tuberculosis* (Mbt) infection is initiated by inhalation into the alveolus. Although studies in lungs of aerosol-infected mice show extensive replication of Mbt in alveolar epithelial cells and other non-macrophage cells and dissemination to other organs early in infection (Ryndak et al., 2015), alveolar MPs are the typical host cells of Mbt where the bacteria primarily reside in phagosomes. Fatty acids and cholesterol are considered to be the most important carbon sources for optimal growth and persistence of Mbt during infection (Lovewell et al., 2016). These two nutrients are involved in the production of energy but also of intermediates for mycolic acids, the major and specific lipid components of the cell envelope of Mbt (Marrakchi et al., 2014). Mbt is also able to utilize several other substrates depending on the growth conditions (Marrero et al., 2013; Beste et al., 2013; Ehrt et al., 2015; Hampel et al., 2015; Rücker et al., 2015) Glucose has been reported to be used for growth *in vivo* and both, glycolysis and gluconeogenesis, are important pathways for the intracellular carbon metabolism (Ehrt et al., 2015).

Not surprisingly, Mbt significantly reprograms the metabolism of the infected host MPs. Metabolomic profiling in mice infected with Mbt showed increased levels of several metabolites in the lung (presumably within MPs) of infected mice which suggest elevated ß-oxidation of lipids and glyoxylate shunting, increased nucleotide and amino acids metabolism and increased anti-oxidative stress response in this tissue upon Mbtinfection (Shin et al., 2011). A hallmark of mycobacterial infection is the formation of foamy MPs characterized by the accumulation of large lipid bodies. The foamy phenotype appears to be associated with the temporary increase of the peroxisome proliferator-activated receptor gamma (PPAR-y) and dependent on TLR-2 (Almeida et al., 2012). It was further shown (Singh et al., 2012) that Mbt induces the foamy phenotype by diverting the glycolytic pathway of the host MPs toward ketone body synthesis. This metabolic dysregulation leads to the activation of the anti-lipolytic G protein-coupled receptor GPR109A which causes perturbations in lipid homeostasis and consequent accumulation of lipid bodies in the MP. ESAT-6, a secreted virulence factor of Mbt interacting with TLR2, seems to be responsible for this feedback loop. The increased MP glycolysis and the accompanied lipid droplet accumulation is in line with the observation that liver X transcriptional receptors (LXRA and LRXB) which control fatty acid, cholesterol and glucose homeostasis are critical regulators of lipid metabolism during intracellular Mbt replication (Han et al., 2014).

### Autophagy may support intracellular replication of cytosol-adapted IBPs

Autophagy is a catabolic process that sequesters and degrades dispensible cellular contents, like waste proteins and damaged organelles, but also microorganisms reaching the cytosol (reviewed by Gomes and Dikic, 2014; Randow and Youle, 2014). Induction of autophagy occurs in response to depletion of ATP, amino acids and glucose and is controlled mainly by AMPK and mTORC1. As outlined above, AMPK is activated by high AMP/ATP ratios while mTORC1 activity is modulated by amino acid levels (Bar-Peled et al., 2012; Inoki et al., 2012).

In the autophagic process, the targeted cytosolic components are engulfed in double membrane surrounded autophagosomes which then fuse with lysosomes leading to the degradation of the autosomal contents by digestive enzymes. This process results in the release of metabolites (in particular amino acids and carbohydrates) that can be reused for anabolic and catabolic processes, especially for protein biosynthesis and energy generation.

“Prosurvival autophagy” may thus promote cell survival under nutrient (especially amino acid) starvation conditions (Dibble and Manning, 2013; Jewell and Guan, 2013), but it may also support microbial replication. Manipulation of both, the energy-sensing AMPK and the nutrient-sensing mTORC1 kinase activities, has been shown to be crucial for the proliferation of several viral pathogens (Brunton et al., 2013); the resulting metabolic changes in the host cells which support viral replication have, however, hardly been analyzed.

The significance of these two key regulators of cell metabolism for the intracellular replication of bacterial pathogens has also been discussed and the importance of autophagy for intracellular growth (through the supply of additional nutrients) has been clearly demonstrated for *Franciscella tularensis* (Steele et al., 2013).

Other IBPs adapted to intracellular life in the host cells cytosol, like *L. monocytogenes* and *Shigella flexneri*, also induce autophagy in MPs (Mostowy et al., 2011). These IBPs have developed specific mechanisms which protect themselves from the autophagic attack. *L. monocytogenes* protects itself by ActA and InlK (Yoshikawa et al., 2009; Dortet et al., 2012) while *S. flexneri* escapes autophagy mainly by IcsB (Ogawa et al., 2005). Autophagy induced in the host cells infected by these IBPs may therefore provide additional nutrients, especially amino acids (Grubmüller et al., 2014) to these intracellular bacteria thus supporting their intracellular replication.

### Other positive effects of metabolically activated immune cells for growth of IBPs

As discussed above, activated NPs infiltrating the intestinal epithelium infected by certain IBPs, e.g., *S. Typhimurium*, will affect the metabolism of the surrounding epithelial cells (e.g., by HIF-1 activation) and, hence, possibly also the replication of IBPs capable of invading these epithelial cells.

Activated NPs may, however, also positively affect the metabolism and proliferation of IBPs in the extracellular state (Winter et al., 2010a; Lopez et al., 2012). The best studied example is *S. Typhimurium* which induces acute inflammation in the intestine by T3SS-1 effectors translocated into epithelial cells and MPs. ROS produced by the attracted activated NPs convert luminal thiosulfate to tetrathionate which serves as *Salmonella*-specific electron acceptor in anaerobic respiration (Winter et al., 2010b). Specific *S. Typhimurium* strains lysogenized by a bacteriophage carrying the *sopE* gene are also able to enhance the production of host-derived nitrate which is an even more efficient electron acceptor than tetrathionate allowing anaerobic nitrate respiration by these bacteria (Lopez et al., 2012). Gut-derived ethanolamine has been suggested as electron donor for this anaerobic respiration chain (Thiennimitr et al., 2011). This metabolic capacity of *S. Typhimurium* (and possibly of other IBPs), supported by activated immune cells, enhances proliferation of these pathogens in the gastrointestinal tract at the expense of the other fermenting resident gut microbes.

The genes essential for this anaerobic metabolism of enteric *S. Typhimurium* appear to be part of a metabolic network which is decaying in the extraintestinal typhoid *S*. Typhi (Nuccio and Bäumler, 2014). *S*. Typhi tightly regulates the expression of T3SS-1 by the regulatory protein TviA, thereby suppressing the pro-inflammatory host responses which reduces intestinal inflammation (Winter et al., 2014) and even impairs activation and proliferation of naïve flagellin–specific CD4^+^ T cells in Peyer's patches and mesenteric lymph nodes (Atif et al., 2014). This allows increased bacterial dissemination finally leading to systemic infection.

Other metabolites produced by activated immune cells in excess, e.g., TCA intermediates like succinate, malate (due to enhanced glutaminolysis) and especially lactate (due to enhanced glycolysis) could be used by IBPs as additional catabolic carbon sources in bipartite metabolism during intracellular replication.

## Conclusions

Immune cells, especially those belonging to the innate branch of the immune system, may function, on the one hand, as powerful defense weapons against microbial pathogens (including IBPs) and, on the other hand, as important host cells for IBPs. These two conflicting functions, executed by the same group of immune cells require specific metabolic programs to which both partners seem to contribute. There is substantial information on the mechanisms leading to the antimicrobial activities of these immune cells that are mainly triggered by the interaction of well-characterized bacterial components with their respective cell receptors. There is also growing knowledge on the various countermeasures which the IBPs have evolved to counteract these antimicrobial cell activities. However, rather little is known how the metabolism of the IBPs has to adapt to that of these host cells in order to achieve optimal intracellular bacterial growth. Even less is known how the metabolism of the immune cells is adjusted to serve either as an antimicrobial environment or as a comfortable replication niche for IBPs. The still quite limited number of studies (broadly discussed in this review) addressing these intriguing problems suggests that different, IBP-specific adaptation strategies are pursued when the pathogens encounter these target cells. This is not unexpected considering the different metabolic potentials of the IBPs and hence their varying nutritional dependency on the host cells, as well as the different arsenal of weapons developed by the IBPs (effector proteins and other virulence factors). A major problem also arises by the heterogeneity of the immune cells of the IIS, especially of MPs (Guilliams et al., 2014; Murray et al., 2014) (for more details, see Supplementary Material), which are often the initial host cells for IBPs during infection. In most reports describing the metabolic reprogramming induced in these cells (even when using primary MPs) it remains unclear to which subtype the actual IBP replication supporting host cell belongs. *In vivo* infection studies, combined with single cell analysis and sensitive techniques allowing the determination of the metabolism in the infected single cells might be necessary to unravel this unsolved problem. The answer to these open questions will not only widen our basic understanding of IBP infections, but may also reveal novel approaches to combat the often severe diseases caused by these pathogens (Bettencourt and Powell, 2017).

## Author contributions

WG and WE designed the study. WG, WE, TR, and JH wrote to article.

### Conflict of interest statement

The authors declare that the research was conducted in the absence of any commercial or financial relationships that could be construed as a potential conflict of interest.

## Footnotes

^1^More recently called “microbe-associated molecular pattern” (MAMP).

^2^According to a new nomenclature based on ontology (for details, see also Supplementary Material), the assignment of this DC subtype has to be revised.

## Abbreviations

4E-BP: factor 4E binding protein
6PG: 6-phosphogluconate
6PGDH: 6-phosphogluconate dehydrogenase
6PGL: 6-phosphogluconolactone
6PGLase: 6-phosphogluconolactonase
ACL: ATP-dependent citrate lyase
AIS: adaptive immune system
AMPK: AMP-activated protein kinase
α-OXO: α-oxoglutarate
BMDM: bone marrow-derived MP
ChREBP: carbohydrate-responsive element-binding protein
CIT: citrate
COX: cytochrome C oxidase
CS: citrate synthase
DAMP: danger-associated molecular patterns
DC: dentritic cell
DHAP: dihydroxyacetone phosphate
E4P: erythrose-4-phosphate
ESX: ESAT-6 secretion system
ETC: electron transport chain
F26BP: fructose-2,6-diphosphate
F6P: fructose-6-phosphate
FBA: fructobisphosphate aldolase
FBP: fructose-1,6-diphosphate
FBPase: fructose-1,6-diphosphatase
FUM: fumarate
FUMH: fumarate hydratase
G-: Gram-negative
G+: Gram-positive
G3P: glyceraldehyde-3-phosphate
G6P: glucose-6-phosphate
G6PDH: glucose-6-phosphate dehydrogenase
GAP: glyceraldehyde-3P
GDH: glutamate dehydrogenase
GLNLY: glutaminolysis
GL: glycolytic pathway
GLS: glutaminase
GLUT: glucose transporter
GLUT1: glucose transporter 1
GN: gluconeogenesis
HGF: hepatocyte growth factor
HIF-1: hypoxia-inducible transcription factor 1
HK2: hexokinase 2
IBP: intracellular bacterial pathogen
ICIT: isocitrate
IDH: isocitrate dehydrogenase
IFN: type-I interferon
IIS: innate immune system
IL: interleukin
InlB: internalin B
iNOS: inducible NO synthase
IRF: interferon regulatory factors
LDH: lactate dehydrogenase
LKB1: liver kinase B1
LPS: lipopolysaccharide
LT: lymphoid tissue
LTA: lipoteichoic acid
MAL: malate
MAMP: microbe-associated molecular pattern
MAPK: mitogen-activated protein kinase
Mbt: *Mycobacterium tuberculosis*
mDAP: meso-diaminopimelate
MDH: malate dehydrogenase
ME: malic enzyme
MEC: mucosal epithelial cell
MO: monocyte
MP: macrophage
MPS: mononuclear phagocyte system
mTOR: target of rapamycin
mTORC1: target of rapamycin complex 1
mTORC2: target of rapamycin complex 2
NLR: (NOD)-like receptor
NLT: non-lymphoid tissue
NOD: nucleotide-binding oligomerization domain
NP: neutrophile
OAA: oxaloacetate
OGDH: oxoglutarate dehydrogenase
OXPHOS: oxidative phosphorylation
PAMP: pathogen-associated molecular pattern
PCK: PEP carboxykinase
PDH: pyruvate dehydrogenase
PDK: pyruvate dehydrogenase kinase
PDPK1: 3-phosphoinositide dependent protein kinase-1
PEP: phosphoenolpyruvate
PFK: phosphofructokinase
PFK1: phosphofructokinase 1
PGI: phosphoglucoisomerase
PGN: peptidoglycan
PHD: prolyl hydroxylase
PI3K: phosphoinositide-3-kinase
PIP2: phosphatidylinositol-4,5-bisphosphate
PIP3: phosphatidylinositol-3,4,5-triphosphate
PK: pyruvate kinase
PKM1: pyruvate kinase M1
PKM2: pyruvate kinase M2
PMN: neutrophilic polymorphonuclear leukocytes
PPAR: peroxisome proliferator-activated receptor
PPP: pentose phosphate pathway
PPS: PEP synthetase
PRR: pattern recognition receptor
PTEN: phosphatase and tensin homolog
PTS: PEP-dependent transport system
R5P: ribose-5-phosphate
RAS: Ras protein
RB: retinoblastoma protein
RIG.I: retinoic acid-inducible gene I
RLR: (RIG.I)-like receptor
RNI: reactive nitrogen intermediate
ROS: reactive oxygen species
RPE: ribulose-5-phosphate 3-epimerase
RPI: ribose-5-phosphate isomerase
Ru5P: ribulose-5-phosphate
S6K: S6 kinase
S7P: sedoheptulose-7-phosphate
SCO2: cytochrome c oxidase assembly protein 2
SDH: succinate dehydrogenase
SHPK: sedoheptulose kinase
SK: skin keratinocyte
SREBP: sterol regulatory element-binding protein
SUC: succinate
T3SS: type 3-secretion system
T4SS: type 4-secretion system
T7SS: type 7-secretion system
TAL: transaldolase
TCA: tricarboxylic acid cycle
TIGAR: TP53-inducible glycolysis and apoptosis regulator
TKT: transketolase
TLR: Toll-like receptor
TNF: tumor necrosis factor
TRAF6: tumor necrosis factor receptor-associated factor 6
X5P: xylulose-5-phosphate.
