# Supplementary material for: To Eat and to Be Eaten: Mutual Metabolic Adaptations of Immune Cells and Intracellular Bacterial Pathogens upon Infection
Source: Front Cell Infect Microbiol. 2017 Jul 13;7:316. doi: 10.3389/fcimb.2017.00316 (PMC5508010; doi:10.3389/fcimb.2017.00316)
Supplement: Supplementary file 1 [file Presentation1.PDF]

Supplementary Material: Future Directions

The classical mononuclear phagocytic system comprises monocytes (MOs)/macrophages (MPs) and dendritic cells (DCs) which can be derived from bone marrow (BM) cells by applying specific culture conditions (Lutz et al., 1999; Martinez and Gordon, 2014). Originally, it was suggested that MOs and DCs are derived from a common MO/DC precursor of BM cells and after terminal differentiation in cell culture they can be distinguished by phenotypic markers and functional properties[1]. However, because of overlapping markers, this kind of cell differentiation proved to be difficult: e.g. the DC marker CD11c can also be expressed by a subgroup of MOs (Drutman et al., 2012). Moreover, activating MO/MP or DC cultures by cytokines, growth factors or microbial stimulants (e.g. PAMPs) may increase the heterogeneity of cell types and activation state. Thus, comparison of metabolomic data of infected or non-infected differentiated BM cells have to consider the cell cultivation protocol, the genotype of the mouse providing the BM cells and the grade of heterogeneity of the differentiated BM cell culture population:

Commonly applied cell culture differentiation protocols for bone marrow cell cultures make use of the plasticity of common DC precursors (CDPs) and common monocyte precursors (MDCs), but ignore the heterogeneity of the selected cell population (Martinez and Gordon, 2014; Mildner and Jung, 2014; Durai and Murphy, 2016):

(i) GM-CSF supplemented medium supports selection of MO-derived DC-like cells (moDC, CD11c^{+}) which comprise a heterogenous population of MPs and cDCs.

(ii) BM culture, supplemented with the DC-specific growth factor FLT3L, results in enrichment of different DC subsets (pDCs, cDC1 and cDC2).

(iii) GM-CSF plus FLT3L selects for CD103^{+} CDs (cDC1).

(iv) M-CSF-supplemented MO cell culture gives rise to MP-like cells (moMP).

(v) M-CSF plus IFN-γ supports differentiation into type 1 MPs (M1) with inflammatory and microbicidal capacity (e.g. expressing inducible nitric oxide synthase i-NOS and production of NO).

(vi) M-CSF plus IL-4 favors differentiation of alternatively activated type 2 MPs (M2) with anti-inflammatory and tissue repair capacity (prevention of NO production by expression of arginase).

Recent studies on the ontogeny of MO/MP and DCs demonstrated that resident tissue MPs of lymphoid and non-lymphoid tissue originate from yolk sac MPs which populate these tissues during embryonic development and maintain themselves by self-renewal after birth (Ginhoux and Guilliams, 2016). This is not applicable for intestinal tissue, where embryo-derived MPs are replaced by extravasated Ly6C^{+}-blood MOs derived from common MO progenitors (cMOP) of the bone marrow. These MOs differentiate to gut MPs (characteristic surface markers: Ly6C^{-}, F4/80^{+}, CD11c^{+}, CX3CR1^{+} and CD64^{+}).

Moreover, cell-type specific transcriptional profiling of single cells, gene depleted mouse models and lineage-tracing reporter models resulted in a change of the conceptual framework of the mononuclear phagocytic system (MPs comprising MOs and DCs). This novel concept is based on CDPs and CMPs derived from hematopoetic stem cells of the bone marrow and allows an improved characterization of the cell subsets in BM-derived cultures.

---

[1] Most of the work cited in this review still relies, as far as the used BM-derived phagocytes are concerned, on this classic nomenclature.

In mice, CDP give rise to three main subtypes (Dalod et al., 2014; Mildner and Jung, 2014; Minarrieta et al., 2017): Classical type 1 DCs (cDC1:XCR1^{+}, CD8α^{+} or XCR1^{+}, CD103^{+} DCs), classical type 2 DCs (cDC2: XCR1^{-}, CD11b^{+}) and plasmacytoid DCs (pDC: CD11c^{int}, CD11b^{-}, B220^{+}). These DC subsets express a cell type specific TLR pattern (TLR3 for cDC1; TLR2, 4, 5 for cDC2 and TLR7, 9 for pDC).
CMP give rise to classical MOs (Ly6C^{hi}) and non-classical MOs (Ly6C^{low}) in the circulation: Ly6^{hi} MO-derived cells (MDCs) extravasate into inflammatory tissue and acquire either DC-like or MP-like properties (Ginhoux and Jung, 2014; Martinez and Gordon, 2014; Murray et al., 2014).

Probably, some of the drawbacks of classical BM cell culture protocols can be avoided in the near future, because BM lineage tracing tools will be available to separate the differentiated BM cells of interest (e.g. by cell sorting) and to develop more specific cell culture protocols for BM derived cells.

References

Dalod, M., Chelbi, R., Malissen, B., and Lawrence, T. (2014). Dendritic cell maturation: functional specialization through signaling specificity and transcriptional programming. *EMBO J* 33**,** 1104-1116.

Drutman, S.B., Kendall, J.C., and Trombetta, E.S. (2012). Inflammatory spleen monocytes can upregulate CD11c expression without converting into dendritic cells. *J Immunol* 188**,** 3603-3610.

Durai, V., and Murphy, K.M. (2016). Functions of Murine Dendritic Cells. *Immunity* 45**,** 719-736.

Ginhoux, F., and Guilliams, M. (2016). Tissue-Resident Macrophage Ontogeny and Homeostasis. *Immunity* 44**,** 439-449.

Ginhoux, F., and Jung, S. (2014). Monocytes and macrophages: developmental pathways and tissue homeostasis. *Nat Rev Immunol* 14**,** 392-404.

Lutz, M.B., Kukutsch, N., Ogilvie, A.L., Rossner, S., Koch, F., Romani, N., and Schuler, G. (1999). An advanced culture method for generating large quantities of highly pure dendritic cells from mouse bone marrow. *J Immunol Methods* 223**,** 77-92.

Martinez, F.O., and Gordon, S. (2014). The M1 and M2 paradigm of macrophage activation: time for reassessment. *F1000Prime Rep* 6**,** 13.

Mildner, A., and Jung, S. (2014). Development and function of dendritic cell subsets. *Immunity* 40**,** 642-656.

Minarrieta, L., Ghorbani, P., Sparwasser, T., and Berod, L. (2017). Metabolites: deciphering the molecular language between DCs and their environment. *Semin Immunopathol* 39**,** 177-198.

Murray, P.J., Allen, J.E., Biswas, S.K., Fisher, E.A., Gilroy, D.W., Goerdt, S., Gordon, S., Hamilton, J.A., Ivashkiv, L.B., Lawrence, T., Locati, M., Mantovani, A., Martinez, F.O., Mege, J.L., Mosser, D.M., Natoli, G., Saeij, J.P., Schultze, J.L., Shirey, K.A., Sica, A., Suttles, J., Udalova, I., Van Ginderachter, J.A., Vogel, S.N., and Wynn, T.A. (2014). Macrophage activation and polarization: nomenclature and experimental guidelines. *Immunity* 41**,** 14-20.
