# Supplementary material for: To Eat and to Be Eaten: Mutual Metabolic Adaptations of Immune Cells and Intracellular Bacterial Pathogens upon Infection
Source: Front Cell Infect Microbiol. 2017 Jul 13;7:316. doi: 10.3389/fcimb.2017.00316 (PMC5508010; doi:10.3389/fcimb.2017.00316)
Supplement: Supplementary file 2 [file Table1.PDF]

Supplementary Table: List of abbreviations

4E-BP	factor 4E binding protein
6PG	6-phosphogluconate
6PGDH	6-phosphogluconate dehydrogenase
6PGL	6-phosphogluconolactone
6PGLase	6-phosphogluconolactonase
ACL	ATP-dependent citrate lyase
AIS	adaptive immune system
AMPK	AMP-activated protein kinase
α-OXO	α-oxoglutarate
BMDM	bone marrow-derived MP
ChREPB	carbohydrate-responsive element-binding protein
CIT	citrate
COX	cytochrome C oxidase
CS	citrate synthase
DAMP	danger-associated molecular patterns
DC	dentritic cell
DHAP	dihydroxyacetone phosphate
E4P	erythrose-4-phosphate
ESX	ESAT-6 secretion system
ETC	electron transport chain
F26BP	fructose-2,6-diphosphate
F6P	fructose-6-phosphate
FBA	fructobisphosphate aldolase
FBP	fructose-1,6-diphosphate
FBPase	fructose-1,6-diphosphatase
FUM	fumarate
FUMH	fumarate hydratase
G-	Gram-negative
G+	Gram-positive
G3P	glyceraldehyde-3-phosphate
G6P	glucose-6-phosphate
G6PDH	glucose-6-phosphate dehydrogenase
GAP	glyceraldehyde-3P
GDH	glutamate dehydrogenase
GLNLY	glutaminolysis
GLP	glycolytic pathway
GLS	glutaminase
GLUT	glucose transporter
GLUT1	glucose transporter 1
GN	gluconeogenesis
HGF	hepatocyte growth factor
HIF-1	hypoxia-inducible transcription factor 1
HK2	hexokinase 2
IBP	intracellular bacterial pathogen
ICIT	isocitrate
IDH	isocitrate dehydrogenase
IFN	type-I interferon
IIS	innate immune system

IL	interleukin
InlB	internalin B
iNOS	inducible NO synthase
IRF	interferon regulatory factors
LDH	lactate dehydrogenase
LKB1	liver kinase B1
LPS	lipopolysaccharide
LT	lymphoid tissue
LTA	lipoteichoic acid
MAL	malate
MAMP	microbe-associated molecular pattern
MAPK	mitogen-activated protein kinase
Mbt	*Mycobacterium tuberculosis*
mDAP	meso-diaminopimelate
MDH	malate dehydrogenase
ME	malic enzyme
MEC	mucosal epithelial cell
MO	monocyte
MP	macrophage
MPS	mononuclear phagocyte system
mTOR	target of rapamycin
mTORC1	target of rapamycin complex 1
mTORC2	target of rapamycin complex 2
NLR	(NOD)-like receptor
NLT	non-lymphoid tissue
NOD	nucleotide-binding oligomerization domain
NP	neutrophile
OAA	oxaloacetate
OGDH	oxoglutarate dehydrogenase
OXPHOS	oxidative phosphorylation
PAMP	pathogen-associated molecular pattern
PCK	PEP carboxykinase
PDH	pyruvate dehydrogenase
PDK	pyruvate dehydrogenase kinase
PDPK1	3-phosphoinositide dependent protein kinase-1
PEP	phosphoenolpyruvate
PFK	phosphofructokinase
PFK1	phosphofructokinase 1
PGI	phosphoglucoisomerase
PGN	peptidoglycan
PHD	prolyl hydroxylase
PI3K	phosphoinositide-3-kinase
PIP2	phosphatidylinositol-4,5-bisphosphate
PIP3	phosphatidylinositol-3,4,5-triphosphate
PK	pyruvate kinase
PKM1	pyruvate kinase M1
PKM2	pyruvate kinase M2
PMN	neutrophilic polymorphonuclear leukocytes
PPAR	peroxisome proliferator-activated receptor
PPP	pentose phosphate pathway

PPS	PEP synthetase
PRR	pattern recognition receptor
PTEN	phosphatase and tensin homolog
PTS	PEP-dependent transport system
R5P	ribose-5-phosphate
RAS	Ras protein
RB	retinoblastoma protein
RIG.I	retinoic acid-inducible gene I
RLR	(RIG.I)-like receptor
RNI	reactive nitrogen intermediate
ROS	reactive oxygen species
RPE	ribulose-5-phosphate 3-epimerase
RPI	ribose-5-phosphate isomerase
Ru5P	ribulose-5-phosphate
S6K	S6 kinase
S7P	sedoheptulose-7-phosphate
SCO2	cytochrome c oxidase assembly protein 2
SDH	succinate dehydrogenase
SHPK	sedoheptulose kinase
SK	skin keratinocyte
SREBP	sterol regulatory element-binding protein
SUC	succinate
T3SS	type 3-secretion system
T4SS	type 4-secretion system
T7SS	type 7-secretion system
TAL	transaldolase
TCA	tricarboxylic acid cycle
TIGAR	TP53-inducible glycolysis and apoptosis regulator
TKT	transketolase
TLR	Toll-like receptor
TNF	tumor necrosis factor
TRAF6	tumor necrosis factor receptor-associated factor 6
X5P	xylulose-5-phosphate
